# Mutual Repression Enhances the Steepness and Precision of Gene Expression Boundaries

**DOI:** 10.1371/journal.pcbi.1002654

**Published:** 2012-08-30

**Authors:** Thomas R. Sokolowski, Thorsten Erdmann, Pieter Rein ten Wolde

**Affiliations:** 1 FOM Institute AMOLF, Amsterdam, The Netherlands; 2 University of Heidelberg, Institute for Theoretical Physics, Heidelberg, Germany; Princeton University, United States of America

## Abstract

Embryonic development is driven by spatial patterns of gene expression that determine the fate of each cell in the embryo. While gene expression is often highly erratic, embryonic development is usually exceedingly precise. In particular, gene expression boundaries are robust not only against intra-embryonic fluctuations such as noise in gene expression and protein diffusion, but also against embryo-to-embryo variations in the morphogen gradients, which provide positional information to the differentiating cells. How development is robust against intra- and inter-embryonic variations is not understood. A common motif in the gene regulation networks that control embryonic development is mutual repression between pairs of genes. To assess the role of mutual repression in the robust formation of gene expression patterns, we have performed large-scale stochastic simulations of a minimal model of two mutually repressing gap genes in *Drosophila*, *hunchback* (*hb*) and *knirps* (*kni*). Our model includes not only mutual repression between *hb* and *kni*, but also the stochastic and cooperative activation of *hb* by the anterior morphogen Bicoid (Bcd) and of *kni* by the posterior morphogen Caudal (Cad), as well as the diffusion of Hb and Kni between neighboring nuclei. Our analysis reveals that mutual repression can markedly increase the steepness and precision of the gap gene expression boundaries. In contrast to other mechanisms such as spatial averaging and cooperative gene activation, mutual repression thus allows for gene-expression boundaries that are both steep and precise. Moreover, mutual repression dramatically enhances their robustness against embryo-to-embryo variations in the morphogen levels. Finally, our simulations reveal that diffusion of the gap proteins plays a critical role not only in reducing the width of the gap gene expression boundaries via the mechanism of spatial averaging, but also in repairing patterning errors that could arise because of the bistability induced by mutual repression.

## Author Summary

Embryonic development is controlled by spatial patterns of gene expression that seal the fate of each cell in the embryo. However, while development is typically very precise, gene expression is often very noisy. Indeed, a key question in current biology is how embryonic development can be so precise, while the underlying processes are very erratic. To address this question, we have performed extensive stochastic simulations of a canonical motif in the gene regulation networks that drive embryonic development, namely mutual repression between pairs of genes. By studying a minimal model of two mutually repressing genes in the fruitfly *Drosophila*, we show that mutual repression can dramatically enhance both the steepness and precision of the boundaries of the gene-expression domains. Moreover, it can buffer against variations in the protein (morphogen) gradients that provide the positional information to the developing embryo. Lastly, our simulations show that diffusion of the expressed protein not only sharpens the boundaries of the expression domains, but also can repair defects that are formed within these domains.

## Introduction

The development of multicellular organisms requires spatially controlled cell differentiation. The positional information for the differentiating cells is typically provided by spatial concentration gradients of morphogen proteins. In the classical picture of morphogen-directed patterning, cells translate the morphogen concentration into spatial gene-expression domains via simple threshold-dependent readouts [1]–[4]. Yet, while embryonic development is exceedingly precise, this mechanism is not very robust against intra- and inter-embryonic variations [5]–[7]: the spatial patterns of the target genes do not scale with the size of the embryo and the boundaries of the expression domains are susceptible to fluctuations in the morphogen levels and to the noise in gene expression. Intriguingly, the target genes of morphogens often mutually repress each other, as in the gap-gene system of the fruit fly *Drosophila* [8]–[14]. To elucidate the role of mutual repression in the robust formation of gene expression patterns, we have performed extensive spatially-resolved stochastic simulations of the gap-gene system of *Drosophila melanogaster*. Our results show that mutual repression between target genes can markedly enhance both the steepness and the precision of gene-expression boundaries. Furthermore, it makes them robust against embryo-to-embryo variations in the morphogen gradients.

The fruit fly *Drosophila melanogaster* is arguably the paradigm of morphogenesis. During the first 90 minutes after fertilization it is a syncytium, consisting of a cytoplasm that contains rapidly diving nuclei, which are not yet encapsulated by cellular membranes. Around cell cycle 10 the nuclei migrate towards the cortex of the embryo and settle there to read out the concentration gradient of the morphogen protein Bicoid (Bcd), which forms from the anterior pole after fertilization [3]. One of the target genes of Bcd is the gap gene *hunchback* (*hb*), which is expressed in the anterior half of the embryo. In spite of noise in gene expression, the midembryo boundary of the *hb* expression domain is astonishingly sharp. By cell cycle 11, the *hb* mRNA boundary varies by about one nuclear spacing only [15]–[17], while by cell cycle 13 a similarly sharp oundary is observed for the protein level [5], [6], [18]. This precision is higher than the best achievable precision for a time-averaging based readout mechanism of the Bcd gradient [6]. Interestingly, the study of Gregor *et al.* revealed that the Hb concentrations in neighboring nuclei exhibit correlations and the authors suggested that this implies a form of spatial averaging that enhances the precision of the posterior Hb boundary [6]. Two recent simulation studies suggest that the mechanism of spatial averaging is based on the diffusion of Hb itself [19], [20]; as shown analytically in [19], Hb diffusion between neighboring nuclei reduces the super-Poissonian part of the noise in its concentration. In essence, diffusion reduces noise by washing out bursts in gene expression. However, the mechanism of spatial averaging comes at a cost: it tends to lessen the steepness of the expression boundaries.

Bcd induces the expression of not only *hb*, but a number of gap genes, and pairs of gap genes tend to repress each other mutually. Interestingly, repression between directly neighboring gap genes is weak, whereas repression between non-adjacent genes is strong [21]. *hb* forms a strongly repressive pair with *knirps* (*kni*) which is expressed further towards the posterior pole; both genes play a prominent role in the later positioning of downstream pair-rule gene stripes [9]. It has been argued that mutual repression can enhance robustness to embryo-to-embryo variations in morphogen levels [12]–[14] and sharpen a morphogen-induced transition between the two mutually repressing genes in a non-stochastic background [22], [23]. However, mutual repression can also lead to bistability [24]–[28]. While bistablity may buffer against inter-embryo variations and rapid intra-embryo fluctuations in morphogen levels, it may also cause stochastic switching between distinct gene expression patterns, which would be highly detrimental. Therefore, the precise role of mutual repression in the robust formation of gene-expression patterns remains to be elucidated.

**Figure 1:**
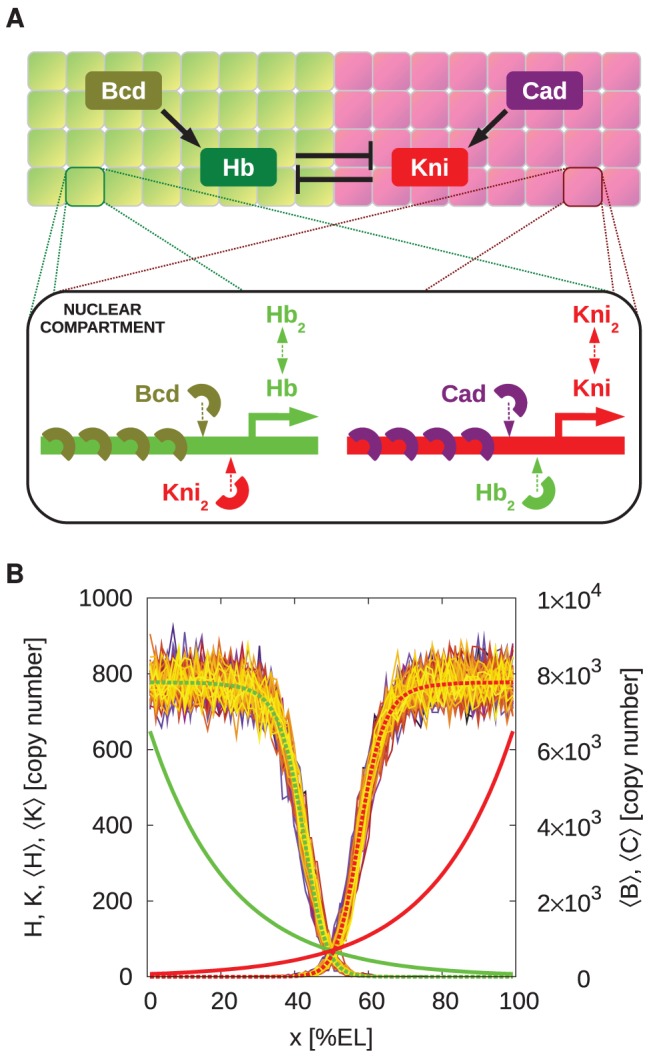
The model. (**A**) Cartoon of our model. Bcd activates *hb*, while its antagonist *kni* is activated by Cad. The gap genes *hb* and *kni* repress each other mutually. In each nuclear compartment we simulate the genetic promoters of both *hb* and *kni*. Activation is cooperative: In the default setting, 5 morphogen proteins have to bind to the promoter to initiate gene expression. Hb and Kni both form homodimers, which can bind to the other gene's promoter to totally block expression, irrespective of the number of bound morphogen proteins. Both dimers and monomers travel between neighboring nuclear compartments via diffusion. (**B**) Protein copy number profiles along the AP axis in a typical simulation in steady state, with parameter values as in Table S2 in Text S1. Plotted are the morphogen gradients Bcd (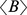, solid green line) and Cad (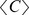, solid red line) and the resulting Hb (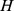) and Kni (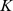) total copy number profiles for different times. The dashed green and red lines show the Hb (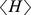) and Kni (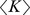) profiles averaged over time and the circumference of the (cylindrical) system.

While the role of antagonistic interactions in the formation of gene-expression patterns has been studied using mean-field models [12], [28]–[31], to address the question whether mutual repression enhances the robustness of these patterns against noise arising from the inherent stochasticity of biochemical reactions a stochastic model is essential. We have therefore performed large-scale stochastic simulations of a minimal model of mutual repression between *hb* and *kni*. Our model includes the stochastic and cooperative activation of *hb* by Bcd and of *kni* by the posterior morphogen Caudal (Cad) [32], [33]. Moreover, Hb and Kni can diffuse between neighboring nuclei and repress each other's expression, generating two separate spatial domains interacting at midembryo (see Fig. 1). We analyze the stability of these domains by systematically varying the diffusion constants of the Hb and Kni proteins, the strength of mutual repression and the Bcd and Cad activator levels. To quantify the importance of mutual repression, we compare the results to those of a system containing only a single gap gene, which is regulated by its morphogen only; this is the “system without mutual repression”. While our model is simplified—it neglects, *e.g.*, the interactions of *hb* and *kni* with *krüppel* (*kr*) and *giant* (*gt*) [34]—it does allow us to elucidate the mechanism by which mutual repression can enhance the robust formation of gene expression patterns.

One of the key findings of our analysis is that mutual repression enhances the robustness of the gene expression domains against intra-embryonic fluctuations arising from the intrinsic stochasticity of biochemical reactions. Specifically, mutual repression increases the precision of gene-expression boundaries: it reduces the variation 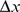 in their positions due to these fluctuations. At the same time, mutual repression also enhances the steepness of the expression boundaries. To understand the interplay between steepness, precision and intra-embryonic fluctuations (biochemical noise), it is instructive to recall that the width 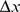 of a boundary of the expression domain of a gene 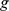 is, to first order, given by
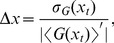
(1)where 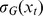 is the standard deviation of the copy number 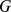 of protein G and 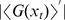 is the magnitude of the gradient of 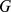 at the boundary position 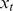 [6], [19], [35]. Steepness thus refers to the slope of the average concentration profile, 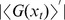, while precision refers to 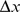, which is the standard deviation in the position at which 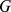 crosses a specified threshold value, here taken to be the half-maximal average expression level of 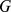.

The simulations reveal, perhaps surprisingly, that mutual repression hardly affects the noise 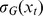 at the expression boundaries of *hb* and *kni*. Moreover, mutual repression can strongly enhance the steepness 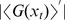 of these boundaries: the steepness of the boundaries in a system with mutual repression can, depending on the diffusion constant, be twice as large as that in the system without mutual repression. Together with Eq. 1, these observations predict that mutual repression can significantly enhance the precision of the boundaries, i.e. decrease 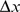, which is indeed precisely what the simulations reveal. Interestingly, there exists an optimal diffusion constant that minimizes the boundary width 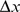, as has been observed for a system without mutual repression [19]. While the minimal 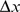 of the system with mutual repression is only marginally lower than that of the system without it, this optimum is reached at a lower value of the diffusion constant, where the steepness of the boundaries is much higher. We find that these observations are robust, i.e. independent of the precise parameters of the model, such as maximum expression level, size of the bursts of gene expression, and the cooperativity of gene activation.

Our results also show that mutual repression can strongly buffer against embryo-to-embryo variations in the morphogen levels by suppressing boundary shifts via a mechanism that is akin to that of [36], [37]. A more detailed analysis reveals that when the regions where Bcd and Cad activate *hb* and *kni* respectively overlap, bistability can arise in the overlap zone. Yet, the mean waiting time for switching is longer than the lifetime of the morphogen gradients, which means that the *hb* and *kni* expression patterns are stable on the relevant developmental time scales. This also means, however, that when errors are formed during development, these cannot be repaired. Here, our simulations reveal another important role for diffusion: without diffusion a spotty phenotype emerges in which the nuclei in the overlap zone randomly express either Hb or Kni; diffusion can anneal these patterning defects, leading to well-defined expression domains of Hb and Kni. Finally, we also study a scenario where *hb* and *kni* are activated by Bcd only. While this scheme is not robust against embryo-to-embryo variations in the morphogen levels, mutual repression does enhance boundary precision and steepness also in this scenario.

## Results

### Model

We consider the embryo in the syncytial blastoderm stage at late cell cycle 14, ca. 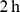 after fertilization. In this stage the majority of the nuclei forms a cortical layer and *hb* and *kni* expression can be detected [11]. Our model is an extension of the one presented in [19]. It is based on a cylindrical array of diffusively coupled reaction volumes which represent the nuclei, with periodic boundary conditions in the angular (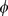) and reflecting boundaries in the axial (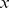) direction. The dimensions of the cortical array are 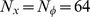, with equal spacing of the nuclei 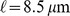 in both directions. For a given embryo length 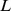, this implies a cylinder radius 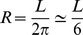, which is close to the experimentally observed ratio. The resulting number of 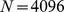 nuclei roughly corresponds to the expected number of cortical nuclei at cell cycle 14 if non-dividing polyploid yolk nuclei are taken into account [38] (see Text S1 for details); we also emphasize, however, that none of the results presented below depend on the precise number of nuclei.

In each nuclear volume we simulate the activation of the gap genes *hb* and *kni* by the morphogens Bcd and Cad, respectively, and mutual repression between *hb* and *kni* (see Fig. 1). In what follows, we will refer to Hb and Kni as repressors and to Bcd and Cad as activators. Our model of gene regulation bears similarities to those of [28], [30], [31], [39], [40], in the sense that it is based on a statistical mechanical model of gene regulation by transcription factors, allowing the computation of promoter-site occupancies. However, the models of [28], [30], [31], [39], [40] are mean-field models, which cannot capture the effect of intra-embryonic fluctuations due to biochemical noise arising from the inherent stochasticity of biochemical reactions. This requires a stochastic model; moreover, it necessitates a model in which the transitions between the promoter states are taken into account explicitly, since these transitions form a major source of noise in gene expression, as we will show. To limit the number of combinatorial promoter states, we have therefore studied a minimal model that only includes Bcd, Cad, Hb and Kni. Following [19], we assume that Bcd and Cad bind stochastically and cooperatively to 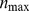 sites on their target promoters. To obtain a lower bound on the precision of the *hb* and *kni* expression domains, we assume that the activating morphogens Bcd and Cad bind to their promoters with a diffusion-limited rate 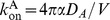, where 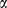 is the dimension of a binding site, 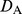 is the diffusion constant of the morphogen, and 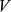 is the nuclear volume (see “Materials & Methods” for parameter values). Since the morphogen-promoter association rate is assumed to be diffusion limited, cooperativity of *hb* and *kni* activation is tuned via the dissociation rate 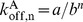, which decreases with increasing number 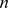 of promoter-bound morphogen molecules. The baseline parameters are set such that the half-maximal activation level of *hb* and *kni* is at midembryo, and the effective Hill coefficient for gene activation is around 5 [19]; while we will vary the Hill coefficient, this is our baseline parameter. Again to obtain a lower bound on the precision of the gap-gene expression boundaries, transcription and translation is concatenated in a single step. Mutual repression between *hb* and *kni* occurs via binding of Hb to the *kni* promoter, which blocks the expression of *kni* irrespective of the number of bound Cad molecules, and vice versa. To assess the importance of bistability, Hb and Kni can homodimerize and bind to their target promoters only in their dimeric form, which is a prerequisite for bistability in the mean-field limit [24]. Both the monomers and dimers diffuse between neighboring nuclei and are also degraded; the effective degradation rate 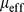 is such that the gap-gene expression domains can form sufficiently rapidly on the time scale of embryonic development (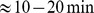 [38]). In the absence of mutual repression, our model behaves very similarly to that of [19], even though our model contains both monomers and dimers instead of only monomers.

Motivated by experiment [3], [5], [7], and in accordance with the diffusion-degradation model, we adopt an exponential shape for the stationary Bcd profile; we thus do not model the establishment of the gradient [41]. To elucidate the role of mutual repression, it will prove useful to take our model to be symmetric: the Cad profile is the mirror image of the Bcd profile, and *hb* and *kni* repress each other equally strongly. Diffusion of Bcd and Cad between nuclei induce fluctuations in their copy numbers on the time scale 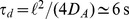. Because 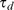 is much smaller than the time scale for promoter binding, 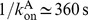, fluctuations in the copy number of Bcd and Cad are effectively averaged out by slow binding of Bcd and Cad to their respective promoters, *hb* and *kni* [19]. To elucidate the importance of the threshold positions for *hb* and *kni* activation, we will scale the morphogen gradients by a global dosage factor 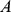; this procedure will also allow us to study the robustness of the system against embryo-to-embryo variations in the morphogen levels.

We simulate the model using the Stochastic Simulation Algorithm (SSA) of Gillespie [42], [43]. Diffusion is implemented into the scheme via the next-subvolume method used in MesoRD [44], [45]. A recent version of our code is available at GitHub and can be accessed via http://ggg.amolf.nl.

### Characteristics of gap-gene expression boundaries

Three key characteristics of gene expression boundaries are 1) the noise in the protein concentration at the boundary; 2) the steepness of the boundary; 3) the width of the boundary. While these quantities may make intuitive sense, their definitions are not unambiguous. Equally important, different definitions will reveal different properties of the system.

#### Decomposing the noise

**Figure 2:**
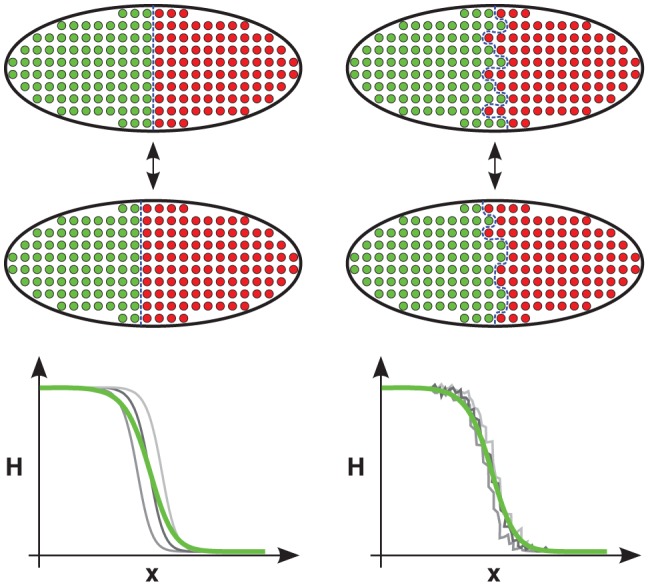
Two different contributions to the intra-embryonic variance in the boundary position. The total variance of the gap gene expression boundary position 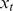 due to intra-embryonic fluctuations, 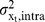, can be decomposed into two contributions: 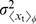, the variance in time of the circumferential mean of 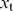, and 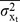, the time-average of the variance of 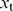 along the circumference of the embryo. The sketch illustrates two extremal cases: If the boundary is very smooth along the circumference at any moment in time, concerted movements of the boundary will dominate the total variance, i.e. 
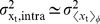
 (left side). If, in contrast, the boundary is rough but its mean position does not fluctuate much in time, then 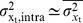 (right side). Naturally, a combination of the two types of fluctuations is possible.

Let's consider the variance in the copy number 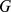 of protein G at position 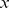 along the anterior-posterior (AP) axis. We define its mean copy number, averaged over all embryos, circumferential positions 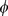 and all times, at the anterior-posterior position 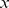 as
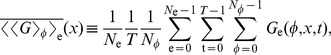
(2)where 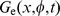 is the copy number of protein G in embryo 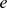 at position 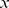 and angle 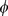 in the circumferential direction (perpendicular to the AP-axis) at time 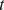. Here, we introduce the convention that the overline denotes an average in time, while the ensemble brackets with a subscript 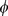 denote an average along the 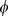 direction and that with a subscript 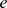 an average over all embryos. The variance in the copy number 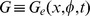 is then given by
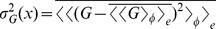
(3)

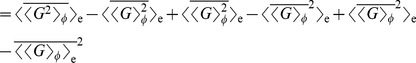
(4)

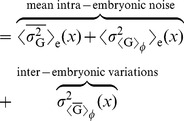
(5)The total variance in the copy number can thus be decomposed into intra-embryonic fluctuations averaged over all embryos and inter-embryonic variations. The former can, furthermore, be decomposed into 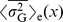, which is the time-averaged mean of the variance in 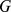 along the circumferential direction, 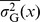, averaged over all embryos, and 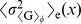, which is the variance in time over the mean of 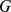 along the circumferential direction, 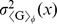, again averaged over all embryos. These intra-embryonic terms capture different types of dynamics. If the expression boundary is rough but its average position does not fluctuate in time, then 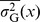 will be large yet 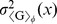 will be small. Conversely, when the boundary is smooth but its average position does fluctuate in time, then 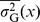 will be small yet 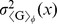 will be large. Naturally, a combination of the two is also possible. The third term, 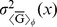, captures the embryo-to-embryo variations in the average over time and 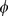 of the protein-copy number. Similarly, we can decompose the fluctuations in the boundary position 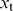 as
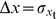
(6)

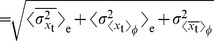
(7)The two different contributions to the intra-embryonic variance, 
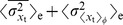
, are illustrated in Fig. 2. Here and in the next section, we will study the robustness of the system against intra-embryonic fluctuations, while in the section “Robustness to inter-embryonic variations: Mutual repression can buffer against correlated morphogen level variations” we will study the robustness against inter-embryonic variations in the morphogen levels.

#### Intra-embryonic fluctuations

Fig. S2 in Text S1 shows the decomposition of the noise in the Hb copy number 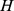 and the threshold position 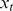 of the Hb boundary, as a function of the diffusion constant. We show the intra-embryonic fluctuations for one given embryo (with the baseline parameter set); how 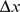 (the boundary variance originating from intra-embryonic fluctuations) changes with embryo-to-embryo variations in the morphogen levels is addressed in section “Overlap of morphogen activation domains does not corrupt robustness to intrinsic fluctuations”. Fig. S2 shows that by far the dominant contribution to the intra-embryonic noise in the copy number and threshold position is the time average of the variance in these observables along the circumferential direction; the variance in time of the 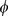-average of these quantities is indeed very small. The picture that emerges is that the expression boundary is rough, even when the diffusion constant 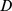 is large, i.e. 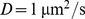. An analysis of the spatial correlation function at midembryo 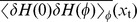, where 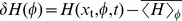, revealed that the correlation length 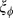 is on the order of a few nuclei, which corresponds to the diffusion length 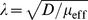 a protein can diffuse with diffusion constant 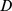 before it is degraded with a rate 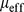; the correlation length is thus small compared to the circumference. One possible source of coherent fluctuations in the mean copy number 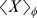 and boundary position 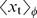 are temporal variations of the morphogen profiles. However, in our model, these profiles are static—we argued that the morphogen fluctuations are fast on the timescale of gene expression, and are thus effectively integrated out. The small correlation length 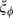 then indeed means that the varations in the mean over 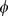, 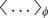, will be small. This leads to an interesting implication for experiments, which we discuss in the Discussion section.

#### The boundary steepness

Now that we have characterized the fluctuations in the copy number and the boundary position, the next question is how fluctuations in the copy number affect the steepness of the boundary. In particular, a gene-expression boundary can be shallow either because at each moment in time the interface is shallow, or because at each moment in time the interface is sharp yet the interface fluctuates in time, leading to a smooth profile. The question is thus how much the gradient of the mean concentration profile, 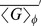, and the mean of the gradient, 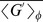, differ (here the prime denotes the spatial derivative). Fig. S3 in Text S1 shows both quantities as a function of the diffusion constant. It is seen that while the average of the gradient is larger than the gradient of the average (as it should), the difference is around a factor of 2. We thus conclude that the steepness of the expression boundary at each moment in time does not differ very much from the steepness of the average concentration profile.

In the rest of the manuscript, we will predominantly focus on the properties of individual embryos, and average quantities are typically averages over time and the circumference. For brevity, therefore, 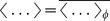, unless stated otherwise.

### Robustness to intra-embryonic fluctuations: Mutual repression allows for steeper profiles without raising the noise level at the boundary

#### Mutual repression shifts boundaries apart

**Figure 3:**
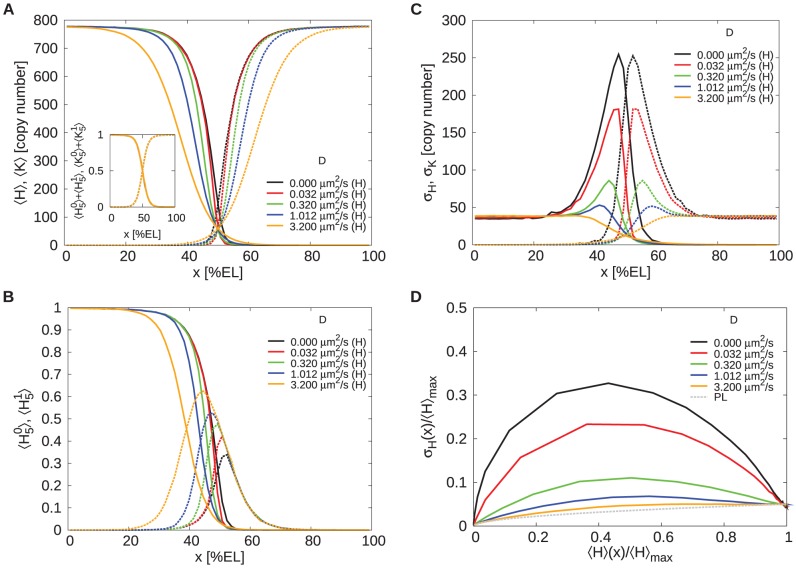
The effect of mutual repression on the average protein concentrations and their standard deviations. (**A**) Time- and circumference-averaged Hb (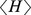, solid lines) and Kni (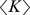, dashed lines) total protein copy number profiles along the AP axis for various diffusion constants 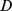 in a system with mutual repression. The inset shows for both the *hb* and the *kni* promoter the probability that the promoter binds 5 morphogen proteins irrespective of whether the antagonistic gap protein is bound to it (meaning that the promoter is activated by the morphogen, even though it may be repressed by the antogonistic gap protein); these “morphogen-activation” profiles are identical for all 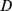 values. (**B**) Profiles of the probability 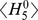 that the *hb* promoter is induced, meaning that it has 5 copies of Bcd bound to it and no Kni dimer (solid lines), and the probability 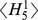 that *hb* is activated by Bcd yet repressed by Kni, in which case *hb* is indeed not expressed (dashed lines). (**C**) AP profiles of the time- and circumference-averaged standard deviation of the total gap protein copy number for Hb (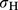, solid lines) and Kni (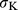, dashed lines). (**D**) Normalized standard deviation 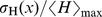 versus the normalized mean 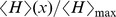; 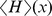 is the averaged total Hb copy number at 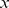 and 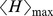 is the maximum of this average over all 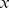. The grey dashed line represents the Poissonian limit (PL) given by 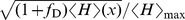, where 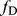 is the fraction of proteins in dimers.

Fig. 3A shows the average Hb and Kni steady-state profiles along the anterior-posterior (AP) axis as a function of their diffusion constant 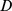 for a system with mutual repression. The inset shows the morphogen-activation profiles, which are the spatial profiles of the probability that the *hb* and *kni* promoters have 5 copies of their respective morphogens bound. Without mutual repression, thus when Hb and Kni cannot bind to their respective target promoters, these profiles describe the probability that *hb* and *kni* are activated by their respective morphogens. Indeed, without mutual repression and without Hb and Kni diffusion, the Hb and Kni concentration profiles would be proportional to their respective morphogen-activation profiles [19], which means that they would precisely intersect at midembryo. In contrast, Fig. 3A shows that the Hb and Kni concentration profiles are shifted apart in the system with mutual repression. There is already a finite separation for 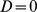, which increases further as 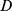 is increased.

In Fig. 3B we show the profile of the probability 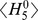 that the *hb* promoter is induced, meaning that it has 5 copies of Bcd bound to it and no Kni, and the profile of the likelihood 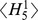 that *hb* is activated by Bcd, yet repressed by Kni, in which case *hb* is not expressed. It is seen that repression by *kni* almost fully inhibits *hb* expression beyond the half-activation point, where *hb* would be expressed without *kni* repression (see inset Panel A). Indeed, mutual repression effectively cuts off protein production beyond midembryo. The production probability therefore changes more abruptly along the AP axis, leading to a higher steepness of the protein profiles near midembryo. For 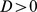, repressor influx over the midplane increases, and as a result the regions of expression inhibiton are enlarged and the concentration profiles shift apart further.

#### Noise reduction via spatial averaging

Fig. 3C shows the standard deviation of the protein copy number along the AP axis for both Hb (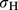) and Kni (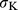). It is seen that the noise increases close to the half-activation point where promoter-state fluctuations are strongest [46]–[48]. This is also observed in Fig. 3D, which shows the normalized standard deviation 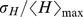 versus the normalized mean 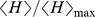 of the average Hb copy number; here, 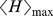 is the maximum average concentration of Hb. The noise maximum close to mid embryo diminishes with increasing 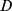, approaching the Poissonian limit. Note that the Poissonian limit here is given by 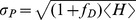, where 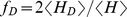 is the fraction of dimerized Hb proteins with respect to the total Hb copy number (see Text S1 for details). Clearly, the spatial averaging mechanism described in [19], [20] reduces the noise also in our system, which differs from those in [19], [20] by the presence of both gap gene monomers and dimers instead of monomers only.

#### Mutual repression reduces the boundary width by increasing the steepness

**Figure 4:**
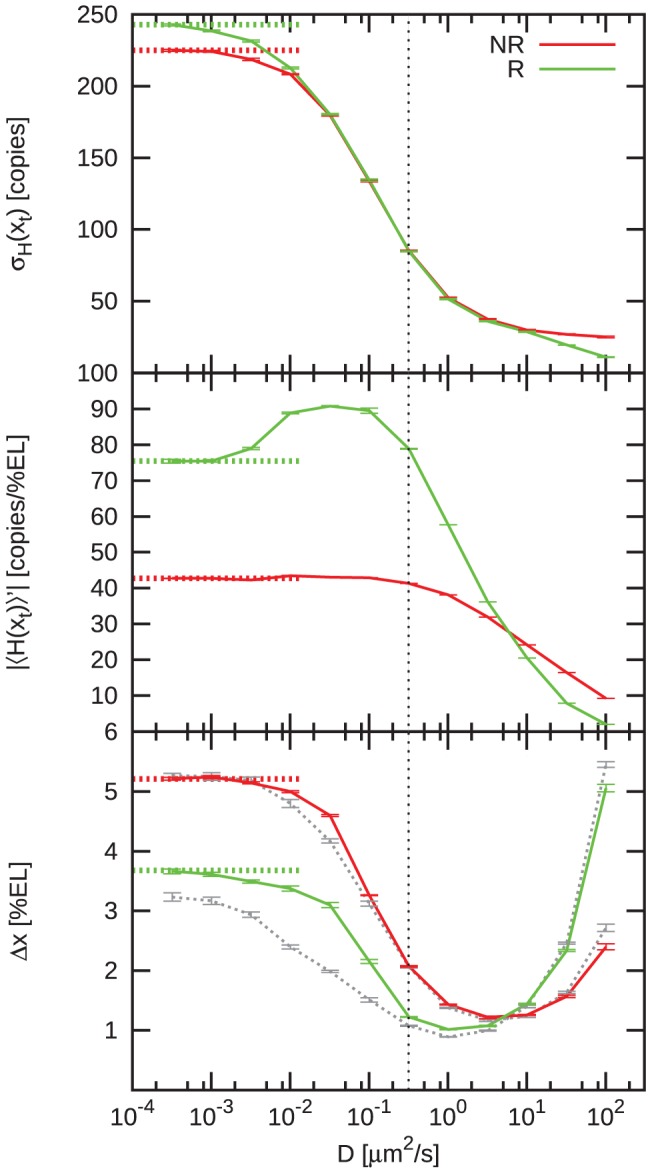
The effect of mutual repression on the precision and steepness of the Hb boundary. The figure shows the time- and circumference-average of the standard deviation of the total Hb copy number at the boundary 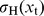 (upper panel), the slope of the total Hb copy number profile at the boundary 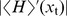 (middle panel) and the Hb boundary width 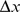 (lower panel) as a function of the diffusion constant 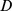 of the gap proteins. Red solid lines show the case without (NR) and green solid lines the case with mutual repression (R); the red and green dashed lines show the limiting values without diffusion of the gap proteins. The grey dashed lines in the boundary width plot are the values based on the approximation 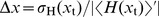. Note that for 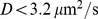, mutual repression enhances the steepness of the boundary, which in turn enhances the precision of the boundary. The black dotted line marks the 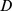-value where the boundary is both steep and precise due to mutual repression.

Fig. 4 quantifies the impact of spatial averaging and mutual repression on the Hb boundary width 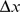, comparing it to that of the system without mutual repression. To first order, the boundary precision 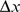 is related to the standard deviation in the protein copy number at the boundary, 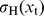, and the steepness of the boundary, 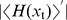, via Eq. 1 [6], [19], [35]. The noise 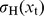 decreases with increasing 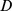 due to spatial averaging in an almost identical manner for the systems with and without mutual repression (Fig. 4, top panel); indeed, perhaps surprisingly, mutual repression has little effect on the noise at the boundary. Increasing 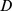 also lessens the steepness of the protein profiles, thus reducing the slope 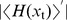 (Fig. 4, middle panel). While without mutual repression this reduction is monotonic, in the case with mutual repression the steepness first rises because increasing 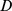 increases the influx of the antagonistic repressor into the regions where the gap genes are activated by their respective morphogens, which, for low values of 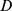, *steepens* the effective gene-activation profile 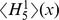 by most strongly reducing gene expression near midembryo; after the steepness has reached its maximum at 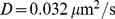, it drops for higher diffusion constants, because the diffusion of the gap-gene proteins now flattens their concentration profiles. Most importantly, with mutual repression 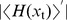 reaches significantly higher values for all 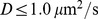. At 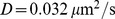 the profile is roughly twice as steep as in the case without repression. Interestingly, for 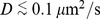, our simulation results for the steepness of the profiles as normalized by their maximal values agree with those measured experimentally by Surkova *et al.* in cell cycle 14 [11]: In both simulation and experiment, the concentration drops from 90% to 10% of the maximal values over 5–10% of the embryo length.

Both with and without Hb - Kni mutual repression the trade-off between noise and steepness reduction leads to an optimal diffusion constant 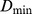 that maximizes boundary precision, i.e. minimizes 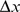 (Fig. 4, lower panel). Mutual repression enhances the precision for 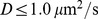 because in this regime decreasing 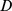 increases the steepness markedly while it has only little effect on the noise as compared to the system without mutual repression. Conversely, 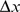 is increased by mutual repression for 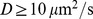 because it reduces the steepness. The minimum in the case with repression is marginally lower than that without (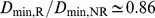), but located at a lower 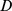-value (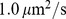 vs. 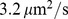). Most importantly, at 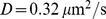, the system with mutual repression produces a profile that is twice as steep as that of the system without it at 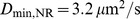, whereas the precision 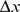 is essentially the same in both cases. Clearly, mutual repression can strongly enhance the steepness of gene-expression boundaries without compromising their precision.

#### Influence of Hill coefficient

A key parameter controlling the precision of the gap-gene expression boundaries, is the degree of cooperativity by which the gap genes are activated by their respective morphogens—this determines the profile steepness of the average gap-gene promoter activity. To investigate this, we have lowered the effective Hill coefficient from its baseline value of 5 by reducing the number 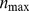 of morphogen molecules that are required to bind the promoter to activate gene expression. To isolate the effect of varying the *mean* gene-activation profiles 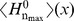 and 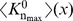, we varied, upon varying 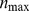, the association and dissociation rates such that 1) the average gene activation probabilities near midembryo, 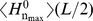 and 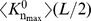, are unchanged and 2) the waiting-time distribution for the gene on-to-off transition is unchanged (since the average activation probability is fixed, the mean off-to-on rate is also unchanged, although the waiting-time distribution is not; see also Fig. S5 in Text S1). We observe that mutual repression markedly enhances the steepness of the gap-gene expression boundaries, also with a lower Hill coefficient for gene activation (Fig. S6 in Text S1). However, lowering the Hill coefficient reduces the steepness of the gene-activation profiles, causing the two antagonistic gene-activation profiles to overlap more. As a result, in each of the two gap-gene expression domains, more of the antagonist is present, which tends to increase the noise in gene expression by occassionally shutting off gene production. This, as explained in more detail later, is particularly detrimental when the diffusion constant is low. Indeed, when the effective Hill coefficient of gene activation is 3 or lower, mutual repression *increases* 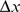 when the diffusion constant is low, i.e. below approximately 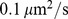. Nonetheless, the *minimal* 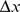 is still lower with mutual repression, and, consequently, also with a lower Hill coefficient for gene activation, mutual repression can enhance both the steepness and the precision of gene-expression boundaries.

#### Influence of the repression strength

**Figure 5:**
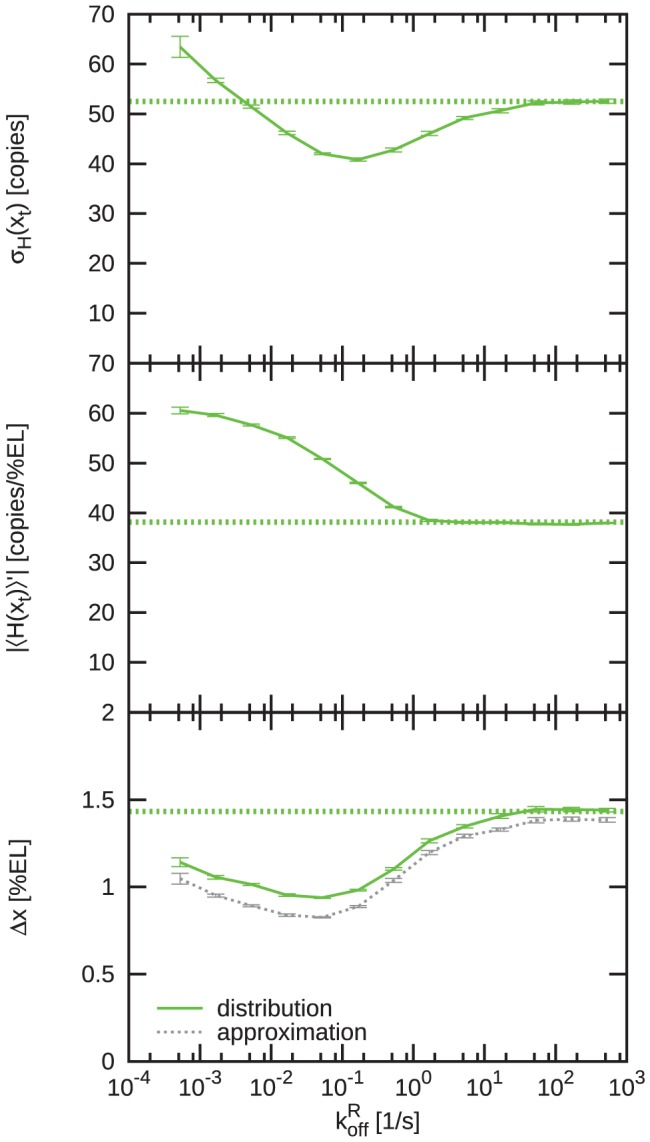
The effect of varying repression strength on the precision and steepness of the Hb boundary. Shown are the time- and circumference average of the standard deviation of the total Hb copy number at the boundary 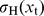 (upper panel), the steepness of the boundary 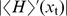 (middle panel) and the Hb boundary width 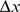 (lower panel) as a function of 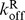, the promoter-dissociation rate of Hb and Kni. The solid green line are values obtained from the boundary position distribution, the dashed grey line the ones calculated from the approximation 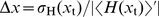. Straight dashed lines mark the limits for the case without mutual repression (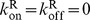).

As a standard we assume very tight binding of the Hb and Kni dimers, “the repressors”, to their respective promoters. To test how this assumption affects our results we performed simulations in which we systematically varied the repressor-promoter dissociation rate 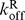 in the range 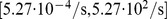, keeping the diffusion constant at 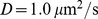 (the value that minimizes the boundary width at 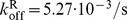) and all other parameters the same as before. Fig. 5 shows the noise, steepness and boundary precision as a function of the repressor-promoter dissociation rate. For high dissociation rates, these quantities equal those in the system without mutual repression (dashed lines). Yet, as the dissociation rate is decreased, the steepness rises markedly at 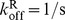. In contrast, the noise 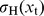 first decreases with decreasing 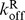, passing through a minimum at 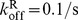 before rising to a level that is higher than that in a system without mutual repression. This minimum arises because on the one hand increasing the affinity of the repressor (the antagonist) makes the operator-state fluctuations of the activator (the morphogen) less important—increasing repressor binding drives the concentration profiles of Hb and Kni away from midembryo, where the promoter-state fluctuations of the activators are strongest; on the other hand, when the repressor binds too strongly, then slow repressor unbinding leads to long-lived promoter states where gene expression is shut off, increasing noise in gene expression; this phenomenon is similar to what has been observed in Refs. [47] and [49], where slower binding of the gene regulatory proteins to the promoter increases noise in gene expression and decreases the stability of a toggle switch, respectively. The interplay between the noise and the steepness yields a marked reduction of the boundary width 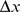; indeed, even in the limit of very tight repressor binding, mutual repression significantly enhances the precision of the boundary.

#### Influence of expression level

Since the precise gap protein expression level is not known, we also varied the maximal protein copy number 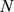 by varying the maximal expression rate 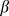 (see Text S1). Fig. S9 in Text S1 shows the output noise and slope at the boundary position, and the boundary precision 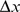, as a function of the diffusion constant for three different expression levels. It is seen that for low diffusion constant, the precision is independent of 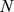, while for higher diffusion constant it scales roughly with 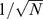. This can be understood by noting that the steepness of the gene-expression boundary scales to a good approximation with 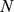 independently of 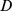, while the noise 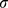 scales with 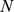 when the diffusion constant is small, but with 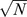 when the diffusion constant is large (see also Eq. 1). The scaling of the noise with 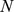 is due to the fact that for low 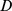 the noise in the copy number is dominated by the noise coming from the promoter-state fluctuations, which scales linearly with 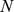, while for high 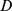, diffusion washes out the expression bursts resulting from the promoter-state flucutations, leaving only the noise coming from the Poissonian fluctuations arising from transcription and translation, which scales with the square root of 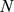 [19]. In Text S1 we also study the importance of bursts arising in the transcription-translation step (see Fig. S8 in Text S1); however, we find that for a typical burst size, these bursts do not dramatically affect boundary precision.

### Robustness to inter-embryonic variations: Mutual repression can buffer against correlated morphogen level variations

Although the Bcd copy number at midembryo has been determined experimentally [6], the measured value is not necessarily the half-activation threshold of *hb*. Indeed, in vivo the Hb profile is shaped by other forces, like mutual repression. In the *kni* - *kr* double mutant, the Hb boundary at midembryo shifts posteriorly [13]. Moreover, gap gene domain formation has been observed at strongly reduced Bcd levels, suggesting that Bcd might be present in excess [50]. Also from a theoretical point of view it is not obvious that a precisely centered morphogen-activation threshold is optimal, in terms of robustness against both intra-embryonic fluctuations and inter-embryonic variations. Here, we study the effect of changing the threshold position where *hb* and *kni* are half-maximally activated by their respective morphogens, Bcd and Cad. While the threshold positions could be varied by changing the threshold morphogen concentrations for half-maximal gap-gene activation (for example by changing the morphogen-promoter dissociation rates), we will vary these positions by changing the amplitude of the morphogen profiles by a factor 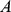. This procedure not only preserves the promoter-activation dynamics at the boundaries—a key determinant for the noise at the boundaries—but also allows us to study the importance of mutual repression in ensuring robustness against embryo-to-embryo variations. Indeed, we will examine not only how changing the threshold position affects the precision of the gap-gene expression boundaries, 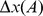, but also how the average boundary positions vary with morphogen dosage, 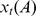, and how the latter gives rise to embryo-to-embryo variations in the boundary position 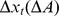 due to embryo-to-embryo variations in the morphogen dosage 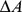.

#### Double-activation induces bistability

We first consider the scenario in which the amplitudes of both morphogens are scaled by the same factor 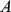. When 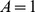, the position at which *hb* and *kni* are half-maximally activated by their respective morphogens coincide at midembryo, meaning that the domains in which *hb* and *kni* are activated beyond half-maximum are adjoining, but do not overlap—this is the scenario discussed in the previous sections. When 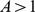, the position at which *hb* is half-maximally activated by its morphogen is shifted posteriorly, while that of *kni* is shifted anteriorly, creating an overlap between the two regions where *hb* and *kni* are activated. In this “double-activated region” both *hb* and *kni* are activated by their respective morphogens, yet they also mutually repress each other. This may lead to bistability. To probe whether this is the case, we performed a bifurcation analysis of the mean-field chemical-rate equations of isolated nuclei, implying that 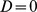 (see Fig. S1 in Text S1). In addition, we performed stochastic simulations of isolated nuclei with different morphogen levels corresponding to different positions along the AP axis. All other parameter values were the same as in the full-scale simulation. We recorded long trajectories of the order parameter 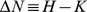, the difference between the total Hb and total Kni copy numbers, in the stationary state. From each trajectory we computed the distribution 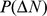 of the probability that the system is in a state with copy number difference 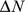. This defines a “free energy” 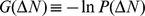, with minima of 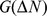 corresponding to maximally probable values of 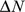 [26], [27]. For a bistable system, 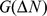 resembles a double-well potential with minima located at a positive value of 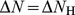 and a negative value of 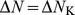, respectively. At midembryo the morphogen levels of Bcd and Cad are the same and hence the biochemical network in the nuclei in the midplane is symmetric, which means that, if this network is bistable, 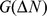 resembles a symmetric double-well potential with 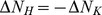 and 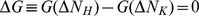. Away from the middle, the morphogen levels differ, and one state will become more stable than the other; if the other state is, however, still metastable, then 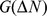 will resemble an asymmetric double-well potential, with 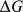 being negative if the *hb* -dominant state is more stable than the *kni* -dominant state, and vice versa. The emergence of such a “spatial switch” along the AP axis is also captured by our mean-field, bifurcation analysis (see Text S1) and was recently also shown in the mean-field analysis of Papatsenko and Levine for the same pair of mutually repressing genes [28].

**Figure 6:**
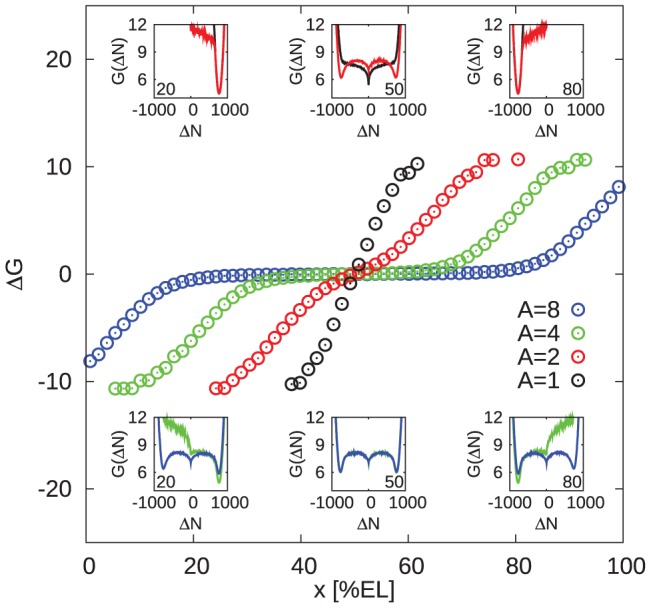
Emergence of bistability in double-activated regions. The “free energy” difference 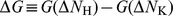 as a function of 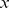, the distance of the nucleus from the anterior pole, for different amplitudes of the morphogen gradients 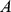; here, 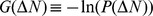, where 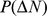 is the stationary distribution of the order parameter 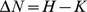; 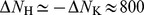 correspond to the minima of 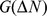. Negative values of 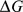 represent a strong bias towards the high- Hb state, while positive values correspond to high- Kni states. The insets shows 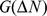 as a function of 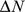 at the positions indicated by the numbers in their corners (values in [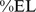]; colors correspond to main plot). The data is obtained from simulations of single nuclei with morphogen levels corresponding to the ones at position 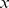 in the full system; this is equivalent to the full system without diffusion between neighboring nuclei. Note the bistable behavior in a wide region of the embryo for higher 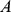 values.

Fig. 6 shows 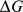 as a function of the position along the AP axis, for different amplitudes 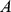 of the morphogen gradients. The inset shows the energy profiles 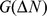 for different positions along the AP axis. For 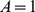, 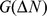 always exhibits one minimum only, irrespective of the position along the AP axis; at midembryo, this minimum is located at 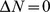, while moving towards the anterior (posterior) the energy minimum rapidly shifts to 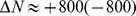, reflecting that in the anterior (posterior) half of the embryo *hb* (*kni*) is essentially fully expressed. For 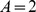, 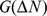 develops into a double-well potential at midembryo, with two pronounced minima at 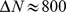 and 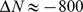, respectively. These two minima correspond to a state in which *hb* is highly expressed (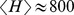) and *kni* is strongly repressed (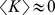) and another state in which *kni* is highly expressed and *hb* strongly repressed, respectively. The fact that the two energy mimima are equal indicates that both of these states are equally likely. Moving away from midembryo, however, one gap-gene expression state rapidly becomes more stable than the other, and bistability is lost, yielding a potential with one minimum located at 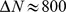 in the anterior half and a potential with one minimum located at 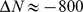 in the posterior half of the embryo. Interestingly, for 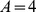 and 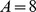 a wide region of bistability develops around midembryo. In this region, 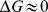, meaning that the high- *hb* —low- *kni* state and the low- *hb* —high- *kni* state are equally stable. These two states are equally likely because in this region both the *hb* and *kni* promoters are fully activated by their respective morphogens. It can also be seen that the width of this bistable region increases with the amplitude of the morphogen gradients, as expected.

#### Slow switching ensures a low noise level while diffusion avoids error locking

The bistability observed for 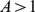 and 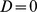 raises an important question, namely whether the nuclei can switch between the two gap-gene expression states on the time scale of embryonic development. This question is particularly pertinent for the higher morphogen amplitudes, where these two states are equally likely (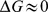) over a wide region of the embryo (Fig. 6): random switching between the two distinct gap-gene expression states in this wide region would then lead to dramatic fluctuations in the positions of the *hb* and *kni* expression boundaries, which clearly would be detrimental for development. We therefore computed [27] from the recorded switching trajectories the average waiting time for switching, 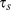, at midembryo (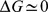) for different values of 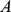; for 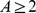, we find 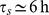 (see Table S1 in Text S1). During cell cycle 14, approximately 2–3 hours after fertilization, the Bcd gradient disappears [51], suggesting that the spontaneous switching rate is indeed low on the relevant time scale of development.

With diffusion of Hb and Kni between neighboring nuclei (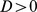), the time scale for switching will be even longer. Diffusion couples neighboring nuclei, creating larger spatial domains with the same gap-gene expression state. This reduces the probability that a nucleus in the overlap region flips to the other gap-gene expression state. The latter can be understood from the extensive studies on the switching behavior of the “general toggle switch” [26], [27], [49], [52]–[54], which is highly similar to the system studied here—indeed, the toggle switch consists of two genes that mutually repress each other. These studies have revealed that the ensemble of transition states, which separate the two stable states, is dominated by configurations where both antagonistic proteins are present in low copy numbers. Clearly, the probability that in a given nucleus not only the minority gap protein, but also the majority gap protein reaches a low copy number, is reduced by the diffusive influx of that majority species from the neighboring nuclei, which are in the same gap-gene expression state. In essence, diffusion increases the effective system size, with its spatial dimension given by 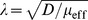; in fact, since the stability of the toggle switch depends exponentially on the system size [26], [27], we expect the stability 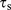 to scale with the diffusion constant as 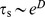. We thus conclude that random switching between the two gap-gene expression states, the high- *hb* —low- *kni* and low- *hb* —high- *kni* states, is not likely to occur on the time scale of early development.

The observation that the switching rate is low raises another important question: if errors are formed during development, can they be corrected? We observe in the simulations with 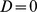 that when we allow the gap-gene expression patterns to develop starting from initial conditions in which the Hb and Kni copy numbers are both zero, in the overlap (bistable) region a spotty gap-gene expression pattern emerges, consisting of nuclei that are either in the high- *hb* —low- *kni* state or in the low- *hb* —high- *kni* state. When the diffusion constant of Hb and Kni is zero, then these defects are essentially frozen in, precisely because of the low switching rate. Interestingly, however, we find in the simulations that a finite diffusion constant *can* anneal these defects. This may seem to contradict the statement made above that diffusion lowers the switching rate. The resolution of this paradox is that while diffusion lowers the switching rate for nuclei that are surrounded by nuclei that are in the same gap-gene expression state, it enhances the switching rate for nuclei that are surrounded by nuclei with a different gap-gene expression state; this is indeed akin to spins in an Ising system below the critical point. The mechanism for the formation of the gap-gene expression patterns, then, depends on the diffusion constant. When 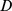 is small yet finite, 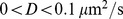, in the overlap region first small domains are formed consisting of nuclei that are in the same gap-gene expression state; these domains then coarsen analogously to Ostwald ripening of small crystallites in a liquid below the freezing temperature; ultimately, they combine with the *hb* or *kni* expression domains that have formed in the meantime outside the overlap region, where *hb* and *kni* are activated by their respective morphogens yet do not repress each other (see Videos S1 and S2). For 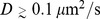, no “crystallites” are formed in the overlap region (both the Hb and Kni copy numbers are low yet finite and *hb* and *kni* simultatenously repress each other); instead, the *hb* and *kni* domains formed near the poles slowly invade the overlap region (see Videos S3 and S4). Interestingly, even while in the absence of Hb and Kni diffusion 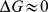 in the overlap region, the interface between the *hb* and *kni* expression domains does slowly diffuse towards midembryo when 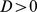 and 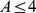, due to the diffusive influx of Hb and Kni from the regions outside the overlap region. When 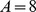, the *hb* and *kni* expression boundaries are not pinned to the middle of the embryo, and their positions exhibit slow and large fluctuations, presumably because the energetic driving force is small, and the diffusive influx of Hb and Kni from the regions near the poles is negligible. We will investigate this effect in more detail in a forthcoming publication.

#### Mutual repression inhibits boundary shifts

**Figure 7:**
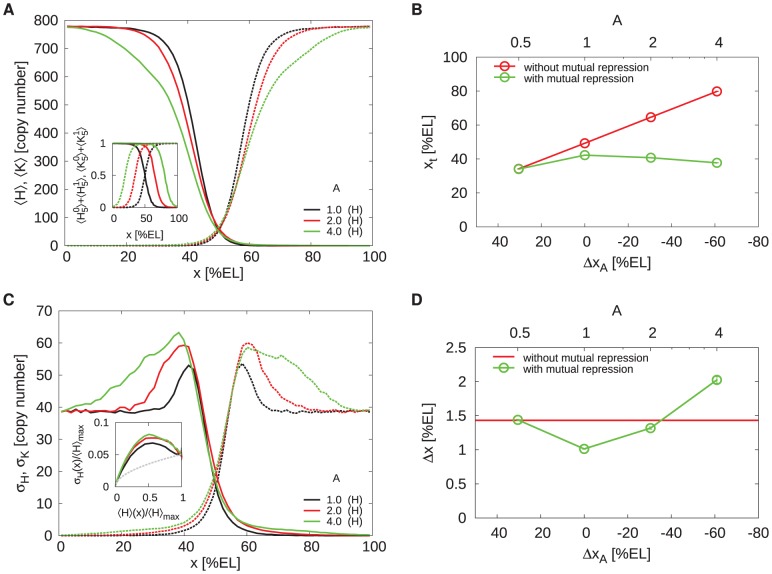
Mutual repression buffers against correlated variations in the activator levels. (**A**) Time- and circumference-averaged Hb (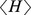, solid lines) and Kni (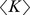, dashed lines) total copy-number profiles along the AP axis for various morphogen dosage factors 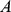. Inset: the corresponding average occupancy of the promoter states with five bound morphogen molecules as a function of 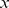. (**B**) The average Hb boundary position 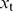 as a function of 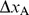, the distance between the Hb and Kni boundaries without mutual repression, for the system with mutual repression (green,) and without it (red); 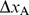 is varied by changing the morphogen dosage factor 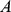. Note that mutual repression makes the gap-gene expression boundaries essentially insensitive to correlated changes in morphogen levels when 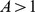. (**C**) AP profiles of the average standard deviation of the total Hb (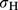, solid lines) and Kni (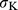, dashed lines) copy numbers. Inset: 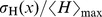 as a function of 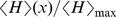, where 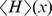 is the average Hb copy number at 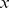 and 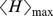 its maximum over 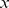. The grey dashed line represents the Poissonian limit. (**D**) The Hb boundary width 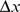 as a function of 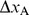 with (green) and without (red) mutual repression. For 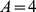, it was impossible to obtain a reliable error bar on 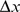, because of the weak pinning force on the *hb* and *kni* expression boundaries.

Fig. 7A shows the average gap-gene expression profiles for 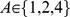 and 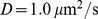, which minimizes the boundary width 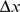 when 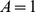 (see Fig. 4). While the morphogen-activation thresholds shift beyond midembryo as 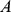 is increased beyond unity, leading to an overlap of the domains where the gap genes are activated by their respective morphogens (see inset), the gap-gene expression boundaries overlap only marginally. This is quantified in panel B, which shows the Hb boundary position 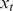 as a function of 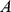 and as a function of 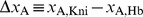, which is defined as the separation between the positions 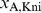 and 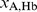 where Kni and Hb are half-maximally activated by their respective morphogens; for 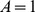, with adjoining morphogen activation regions, 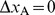 and for 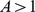, with overlapping activation regions, 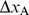 is negative. Without mutual repression (red data), the Hb boundary position 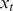 tracks the shift of the *hb* activation threshold, as expected. In contrast, with mutual repression (green data) the boundary does not move beyond the position for 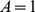 as 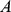 is increased. The same robustness was also observed for other values of the Hill coefficient of gap-gene activation (see Fig. S7 in Text S1).

#### Mutual repression enhances robustness to embryo-to-embryo variations

The fact that mutual repression can pin expression boundaries, dramatically enhances the robustness against embryo-to-embryo variations in the morphogen levels. We did not sample inter-embryo variations in 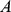 explicitly, but made an estimate using 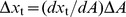, where 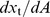 was taken from Fig. 7B. A correlated symmetric variation 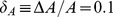 of both morphogen levels then would lead to 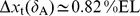 at 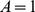 and 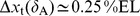 at 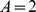. Without mutual repression 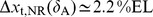. This analysis thus suggests that mutual repression reduces boundary variations due to fluctuations in the morphogen levels by almost a factor of 10 if the half-activation threshold is slightly posterior to midembryo (e.g. 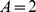). If, on average, 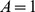, then mutual repression still reduces 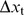 by inhibiting posterior shifts in those embryos in which 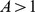. These results are consistent with those of [14], [36].

#### Overlap of morphogen activation domains does not corrupt robustness to intrinsic fluctuations

While mutual repression proves beneficial in buffering against embryo-to-embryo variations in morphogen levels, the question arises whether overlapping morphogen-activation domains does not impair robustness to intrinsic fluctuations arising from noisy gene expression and diffusion of gap gene proteins. We found that this depends on the Hill coefficient of gap-gene activation, which depends on the number 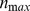 of morphogen binding sites on the promoter. Fig. 7C shows, for 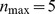, that even though mutual repression increases the noise in gap-gene expression away from the boundaries, it has little effect on the noise at the boundaries when 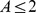. For 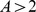, the noise does increase significantly; in fact, it was impossible to obtain reliable error bars, because of the weak pinning force of the *hb* - *kni* interface. Moreover, overlapping morphogen activation domains decrease the steepness of the expression boundaries (panel A), and this increases the boundary width 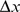 (panel D). Indeed, when 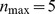, mutual repression can enhance the precision of gene-expression boundaries, but only if the activation domains are adjoining (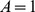), or have a marginal overlap (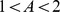). For lower values of 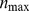, however, this enhancement of precision extends over a much broader range of 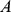 values; in fact, when 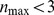, mutual repression enhances precision even up to 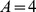 (see Fig. S7 in Text S1).

### Boundaries shift upon uncorrelated variations in morphogen levels, yet intrinsic noise remains unaltered

**Figure 8:**
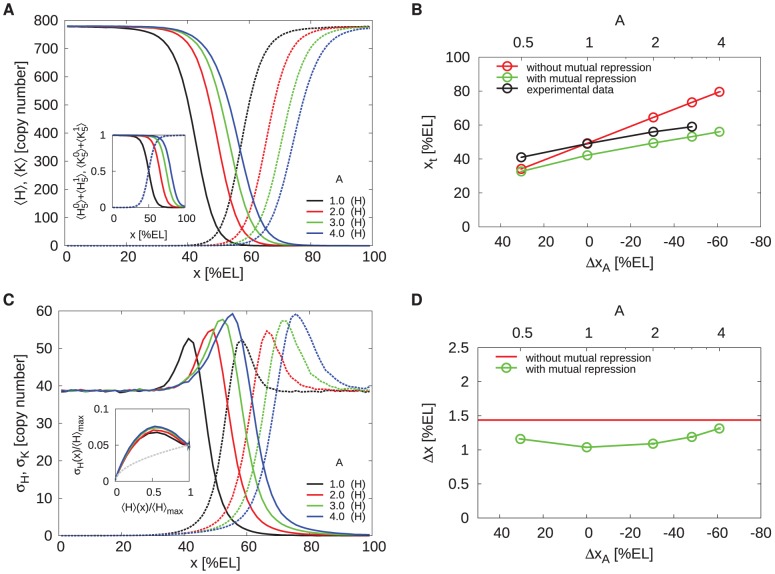
Robustness of the gap-gene expression boundaries to variations in the *bcd* gene dosage. (**A**) Time- and circumference-averaged Hb (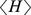, solid lines) and Kni (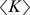, dashed lines) total copy-nymber profiles along the AP axis for various *bcd* gene dosage factors 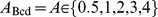 and 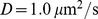. Inset: the average occupancy of the promoter states with five bound morphogen molecules as a function of 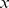. (**B**) Comparison of the boundary position 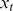 as a function of 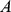 for 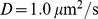 to values measured by Houchmanzadeh et al. [5] (black line). The red line shows the simulation results for the system without mutual repression. Note the good agreement between the experimental data and the simulation data of the system with mutual repression. (**C**) Profiles of the average standard deviation of the total Hb (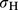, solid lines) and Kni (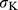, dashed lines) copy number. Inset: 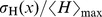 as a function of 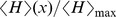. The grey dashed line represents the Poissonian limit. (**D**) The Hb boundary width 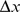 as a function of 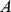 and 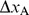, the separation between the Hb and Kni boundaries in a system without mutual repression, for the system with (green) and without (red) mutual repression. 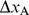 is varied by multiplying the Bcd level by 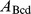.

Since correlated upregulation of both morphogen levels is a special case, we also studied the effect of uncorrelated activator scaling. To this end, only the Bcd level was multiplied by a global factor 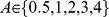, while other parameters were left unchanged. Again we investigated the Hb boundary position 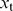, its variance 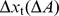 due to extrinsic (embryo-to-embryo) variations in 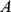 and the variance due to intrinsic (intra-embryo) fluctuations 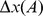. Results for 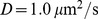 are summarized in Fig. 8.

#### The Hb boundary shifts less with mutual repression

Fig. 8A shows that the *hb* expression boundary shifts posteriorly with increasing 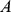, in contrast to the case of correlated activator scaling. The Kni profile retracts in concert with the advance of the Hb domain. In Fig. 8B we compare the Hb boundary 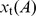 to the data of Houchmandzadeh et al. [5], assuming a 100% efficiency of the additional *bcd* gene copies. It is seen that the agreement between simulation and experiment is very good: while 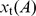 of the simulations has a marginal offset as compared to the experimental data, the slope of 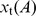 is essentially the same. Moreover, the slope is much lower than that obtained without mutual repression, showing that mutual repression can indeed buffer against uncorrelated variations in morphogen levels. These results parallel those of [36].

#### Robustness to inter-embryo fluctuations

To estimate the boundary variance due to inter-embryo variations in morphogen levels, we fitted a generic logarithmic function 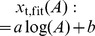 to the simulation data, giving 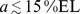 for all values of 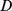 studied. Hence 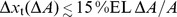. A 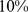 variability in 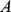 around 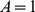 thus would result in 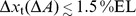, which is half as much as predicted by the model in [36] for that case. Nevertheless, it is yet too large to correspond to the experimental observations of Manu et al. that variations in the Bcd gradient of 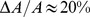 correspond to variations in the Hb boundary position of 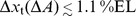 [13]. Our results therefore support their conjecture that higher levels of Bcd are correlated with upregulation of Kni and Cad.

#### Robustness to intra-embryo fluctuations

The output noise at the Hb boundary remains largely unaffected (Fig. 8C and inset) by Bcd upregulation, whereas the slope is reduced by approximately 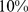 per doubling of 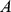 (data not shown). As a result, the boundary width 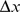 stays close to 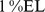 for all considered 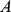 (green data; Fig. 8D), remaining lower than that obtained without mutual repression (red data; Fig. 8D).

### Mutual repression with one morphogen gradient

In the mutual repression motif discussed above, the two antagonistic genes were activated by independent morphogens, one emanating from the anterior and the other from the posterior pole. An alternative mutual repression motif is one in which the two genes are activated by the same morphogen, *e.g. hb* and *kni* both being activated by Bcd [22], [55].

We simulated a system in which *hb* and *kni* mutually repress each other, yet both are activated by Bcd, with *kni* having a lower Bcd activation threshold than *hb*. This generates a Hb and Kni domain, with the latter being located towards the posterior of the former (see Fig. S4 in Text S1). We systematically varied the mutual repression strength and the diffusion constant, to elucidate how mutual repression and spatial averaging sculpt stable expression patterns in this motif. Our analysis reveals that since *hb* and *kni* are both activated by the same morphogen gradient, *hb* should repress *kni* more strongly than vice versa: with equal mutual repression strengths either a spotty gap-gene expression pattern emerges in the anterior half, namely when the Hb and Kni diffusion constant are low (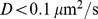), or Kni dominates or even squeezes out Hb, namely when their diffusion constant is large. Nonetheless, for unequal mutual repression strengths and sufficiently high 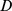, the repression of *hb* by *kni* does enhance the precision and the steepness of the Hb boundary, although the effect is smaller than in the two-gradient motif (Fig. S4 in Text S1). Clearly, while the one-morphogen-gradient motif cannot provide the robustness against embryo-to-embryo variations in morphogen levels that the two-morphogen-gradient motif can provide, mutual repression can enhance boundary precision also in this motif.

## Discussion

Using large-scale stochastic simulations, we have examined the role of mutual repression in shaping spatial patterns of gene expression, with a specific focus on the *hb* - *kni* system. Our principal findings are that mutual repression enhances the robustness both against intra-embryonic fluctuations due to noise in gap-gene expression and embryo-to-embryo variations in morphogen levels.

To investigate the importance of mutual repression in shaping gene-expression patterns, we have systematically varied a large number of parameters: the strength of mutual repression, the diffusion constant of the gap proteins, the maximum expression level, the Hill coefficient of gap-gene activation, and the amplitude of the morphogen gradients. To elucidate how varying these parameters changes the precision of the gap-gene boundaries, we examined how they affect both the steepness of the gene-expression boundaries and the expression noise at these boundaries (see Eq. 1). The effect on the steepness is, to a good approximation, independent of the noise, and would therefore be more accessible experimentally. We find that the steepness increases with decreasing diffusion constant, but increases with increasing strength of mutual repression, maximum expression level, and Hill coefficient of gap-gene activation. Moreover, mutual repression shifts the expression boundaries apart and makes the system more robust to embryo-to-embryo variations in the morphogen levels. In contrast, the noise at the expression boundaries decreases with increasing diffusion constant, decreasing expression level, and decreasing Hill coefficient, while the dependence on the strength of mutual repression is non-monotonic, albeit not very large. The interplay between noise and steepness means that the precision of the gap-gene expression boundaries increases (i.e., 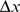 decreases) with increasing expression level. The dependence of 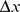 on the diffusion constant and the strength of mutual repression, on the other hand, is non-monotonic: there is an optimal diffusion constant and repression strength that maximizes precision. The effect of the Hill coefficient is conditional on the strength of mutual repression: without mutual repression, the precision slightly decreases with increasing Hill coefficient, while with mutual repression the precision increases with increasing Hill coefficient.

While mutual repression has only a weak effect on the noise in the expression levels at the gene-expression boundaries, it does markedly steepen the boundaries, especially when the diffusion constant is low. Indeed, mutual repression can enhance the precision of gene expression boundaries by steepening them. Nonetheless, even with mutual repression spatial averaging [19], [20] appears to be a prerequisite for achieving precise expression boundaries: without diffusion of the gap proteins, the width of the *hb* expression boundary is larger than that observed experimentally [6]. Hence, while previous mean-field analysis found diffusion not be important for setting up gene-expression patterns [12], [28], our analysis underscores the importance of diffusion in reducing copy-number fluctuations. In addition, diffusion can anneal patterning defects that might arise from the bistability induced by mutual repression. Diffusion is, indeed, a potent mechanism for reducing the effect of fluctuations, such that mean-field analyses can accurately describe mean expression profiles.

Interestingly, the minimum boundary width at the optimal diffusion constant in a system with mutual repression is not much lower than that in one without mutual repression. Yet, in the latter case the boundary width is already approximately one nuclear spacing, and there does not seem to be any need for reducing it further. However, with mutual repression, the same boundary width can be obtained at a lower diffusion constant, where the steepness of the boundaries is much higher, approximately twice as high as that without mutual repression. Our results thus predict that mutual repression allows for gap-gene expression boundaries that are both precise and steep. In fact, the width and steepness of the boundaries as prediced by our model are in accordance with those measured experimentally [11].

Our observation that mutual repression increases the steepness of gene-expression boundaries without significantly raising the noise, makes the mechanism distinct from other mechanisms for steepening gene expression boundaries, such as lowering diffusion constants [19] or increasing the cooperativity of gene activation (see Fig. S6 in Text S1). These mechanisms typically involve a trade off between steepness and noise: lowering the diffusion constant or increasing the Hill coefficient of gene activation steepens the profiles but also raises the noise in protein levels at the expression boundary. In fact, increasing the Hill coefficient (without mutual repression) *decreases* the precision of gene-expression boundaries. This is because increasing the Hill coefficient increases the width of the distribution of times during which the promoter is off, leading to larger promoter-state fluctuations and thereby to larger noise in gene expression (see Fig. S5 in Text S1).

Another important role of mutual repression as suggested by our simulations is to buffer against inter-embryonic variations in the morphogen levels. Houchmandzadeh *et al.* observed that in *bcd* overdosage experiments the Hb boundary does not shift as far posteriorly as predicted by the French flag model [5]. One possible explanation that has been put forward is that Bcd is inactivated in the posterior half of the embryo via a co-repressor diffusing from the posterior pole [36]. More recently, it has been proposed that gap gene cross regulation underlies the resilience of the gap-gene expression domains towards variations in the *bcd* gene dosage [12], [13]. Our analysis supports the latter hypothesis. In particular, our results show that when the regions in which *hb* and *kni* are acitvated by their respective morphogens overlap, the boundary positions are essentially insensitive to correlated variations in both morphogen levels, and very robust against variations of the Bcd level only, with the latter being in quantitative agreement with what has been observed experimentally [5]. Moreover, when this overlap is about 0–20% of the embryo length, mutual repression confers robustness not only against inter-embryonic variations in morphogen levels, but also intra-embryonic fluctuations such as those due to noise in gene expression.

Manu *et al.* found that in the *kr* ; *kni* double mutant, which lacks the mutual repression between *hb* and *kni*/*kr*, the Hb midembryo boundary is about twice as wide as that in the wild-type embryo [13]. This could be due to a reduced robustness against embryo-to-embryo variations in morphogen levels, but it could also be a consequence of a diminished robustness against intra-embryonic fluctuations. The analysis of Manu *et al.* suggests the former [12], [13], and also our results are consistent with this hypothesis. However, our results also support the latter scenario: for 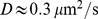, the Hb boundary width in the system without mutual repression is about twice as large as that in the system with mutual repression (see Fig. 4C). Clearly, new experiments are needed to establish the importance of intra-embryonic fluctuations versus inter-embryonic variations in gene expression boundaries.

To probe the relative magnitudes of intra- vs inter-embryonic variations, one ideally would like to measure an ensemble of embryos as a function of time; one could then measure the different contributions to the noise in the quantity of interest following Eq. 5. This, however, is not always possible; staining, e.g., typically impedes performing measurements as a function of time. The question then becomes: if one measures different embryos at a given moment in time, are embryo-to-embryo variations in the mean boundary position or protein copy number (thus averaged over the circumference) due to intra-embryonic fluctuations in time or due to systematic embryo-to-embryo variations in e.g. the morphogen levels? Experiments performed on different embryos but at one time point cannot answer this question. Our analysis, however, suggests that the intra-embryonic fluctuations in the mean copy number or boundary position (i.e. averaged over 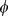) over time are very small, and that hence embryo-to-embryo variations in the mean quantity of interest are really due to systematic embryo-to-embryo variations; these variations then correspond to 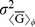 or 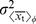 in Eq. 5 or Eq. 7, respectively. The intra-embryonic fluctuations, 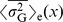 or 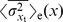, can then be measured by measuring the quantity of interest, 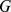 or 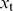, as a function of 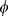, and averaging the resulting variance over all embryos. We expect that these observations, in particular the critical one that intra-embryonic fluctuations in the mean quantity of interest are small, also hold for non-stationary systems, although this warrants further investigation.

Our model does not include self-activation of the gap genes. Auto-activation has been reported for *hb*, *kr* and *gt*, but there seems to be no evidence in case of *kni* [34], [56]. The self-enhancement of gap genes has the potential to steepen and sharpen expression domains even more by amplifying local patterns [57], [58]. Our results suggest, however, that auto-activation is not necessary to reach the boundary steepness and precision as observed experimentally.

Our results provide a new perspective on the Waddington picture of development [59], [60]. Waddington argued that development is “canalized”, by which he meant that cells differentiate into a well-defined state, despite variations and fluctuations in the underlying biochemical processes. It has been argued that canalization is a consequence of multistability [12], [13], [28], which is the idea that cells are driven towards attractors, or basins of attraction in state space. To determine whether a given system is multistable, it is common practice to perform a stability analysis at the level of single cells or nuclei. Our results show that this approach should be used with care: diffusion of proteins between cells or nuclei within the organism can qualitatively change the energy landscape; specifically, a cell that is truly bistable without diffusion might be monostable with diffusion. Indeed, our results highlight that a stability analysis may have to be performed not at the single cell level, but rather at the tissue level, taking the diffusion of proteins between cells into account.

Finally, while our results have shown that mutual repression can stabilize expression patterns of genes that are activated by morphogen gradients, one may wonder whether it is meaningful to ask the converse question: do morphogen gradients enhance the stability of expression domains of genes that mutually repress each other? This question presupposes that stable gene expression patterns can be generated without morphogen gradients. Although it was shown that confined (though aberrant) gap gene patterns form in the absence of Bcd [61]–[63] and that Hb can partly substitute missing Bcd in anterior embryo patterning [64], it is not at all obvious how precise domain positioning could succeed in such a scenario. In particular, one might expect that with mutual repression only, thus without morphogen gradients, there is no force that pins the expression boundaries. Our results for the large overlapping morphogen-activation domains, with 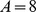, illustrate this problem: in the overlap region, both *hb* and *kni* are essentially fully activated by their respective morphogens, as a result of which the morphogen gradients cannot determine the positions of the gap-gene boundaries within this region; indeed, mutual repression has to pin the expression boundaries of *hb* and *kni*. Yet, our results show that in this case the positions of the *hb* and *kni* expression boundaries exhibit large and slow fluctuations, suggesting that mutual repression alone cannot pin expression boundaries. Interestingly, however, with 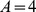, the region in which both genes are activated is still quite large, about 50% of the embryo, and yet even though the underlying energy landscape is flat in this region, the interfaces do consistently move towards the middle of the embryo, due to diffusive influx of Hb and Kni from the polar regions. It is tempting to speculate that mutual repression and diffusion can maintain stable expression patterns, while morphogen gradients are needed to set up the patterns, *e.g.* by breaking the symmetry between the possible patterns that can be formed with mutual repression only.

## Materials and Methods

In the following we describe details of our parameter choice and sampling technique. To unravel the mechanisms by which mutual repression shapes gene-expression patterns, it is useful to take the Cad - Kni -system to be a symmetric copy of the Bcd - Hb -system. Cad thus inherits its parameters from Bcd and Kni from Hb, if not otherwise stated. Table S2 in Text S1 gives an overview of our standard parameter values. Data from experiments was used whenever possible. When it was unavailable we made reasonable estimates.

### Binding rates are diffusion limited

We assume all promoter binding rates to be diffusion limited and calculate them via 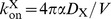. Here 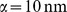 is the typical size of a binding site, 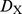 is the intranuclear diffusion constant of species 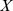 and 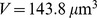 is the nuclear volume. The precise values of 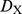 for the different species in our system are not known. Gregor et al. have shown experimentally that the nuclear concentration of Bcd is in permanent and rapid dynamic equilibrium with the cytoplasm [7], suggesting that nuclear and cytoplasmic diffusion constants can be taken for equal. They have found 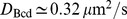 by FRAP measurements. This value has been subject to controversy because it is too low to establish the gradient before nuclear cycle 10 (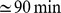) by diffusion and degradation only, prompting alternative gradient formation models [65]–[70]. A more recent study revisited the problem experimentally via FCS, yielding significantly higher values for 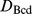 up to 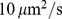 with a lower limit of 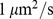 [71]. We therefore have chosen a 10× higher value of 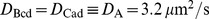 as compared to the earlier choice in [19]. For simplicity, this value is taken for all binding reactions occuring in our model, except for the dimerization reaction rate 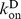, which is taken to be higher by a factor of 2 to account for the fact that both reaction partners diffuse freely.

To model cooperative activation of *hb* and *kni* by their respective morphogens, the morphogen-promoter dissociation rate is given by 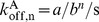, where 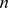 is the number of morphogen molecules that are bound to the promoter; for our standard cooperativity 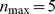 the values of 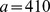 and 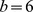 have been chosen such that the threshold concentration for promoter activation (in the absence of repression) equals the observed average number of morphogen molecules at midembryo (when 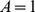, see below). 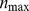 is varied in some simulations; we describe in Text S1 how 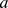 and  are chosen in these cases. The promoter unbinding rate of *hb* and *kni* (the repressor-promoter unbinding rate) 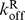 is a parameter that we vary systematically. To study the potential role of bistability we decided to set 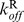 to a value which ensures bistable behavior when both *hb* and *kni* are fully activated by their respective morphogens (meaning that all five binding morphogen-binding sites on the promoter are occupied). This requires tight repression, yielding dissociation constants 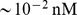 (but see also below). The dimer dissociation rate is set to be 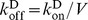, which is motivated by the choice for the toggle switch models studied in [26], [27] and [49], and asserts that at any moment in time the majority of the gap proteins is dimerized. This is a precondition for bistability in the mean-field limit [24], [26], [27].

The parameters of the exponential morphogen gradients are chosen such that the number of morphogen molecules at midembryo and the decay length of the gradient are close to the experimentally observed values for Bcd, 690 and 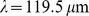, respectively [6].

### Production and degradation dynamics

The copy numbers of both monomers and dimers and the effective gap gene degradation rate 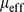 depend in a nontrivial manner on production, degradation and dimerization rates. However, for constant production rate 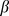, without diffusion and neglecting promoter dynamics, an analytical estimate for the monomer and dimer copy numbers can be obtained from steady state solutions of the rate equations (see Text S1). Based on this we have made a choice for 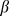 and the monomeric (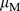) and dimeric (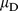) decay rates that leads to reasonable copy numbers and 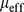 (see Table S2 in Text S1). The latter is defined as the mean of  and 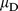 weighted by the species fractions. 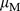 and 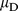 are set such that 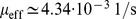, which corresponds to an effective protein lifetime of 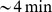. This is close to values used earlier [19], [36] and allows for the rapid establishment of the protein profiles observed in experiments. The dimers have a substantially lower degradation rate than monomers, which enhances bistability [72]. The lower decay rate of the dimers may be attributed to a stabilizing effect of oligomerization (cooperative stability) [72].

### Free parameters

One of the key parameters that we vary systematically is the internuclear gap gene diffusion constant 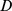, which defines a nuclear exchange rate 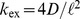 (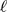 = internuclear distance). To study the effect of embryo-to-embryo variations in the morphogen levels, the latter are scaled globally by a dosage factor 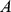. We considered two scenarios: scaling both gradients by the same 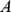 (“correlated variations”) or scaling the Bcd gradient only (“uncorrelated variations”). To test how strongly the assumption of strong repressor-promoter binding affects our results, we also varied the repressor-promoter dissociation rate 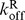. Moreover, to study the dependence of our results on the gap-gene copy numbers, we also increased the protein production rate 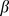. These simulations are much more computationally demanding; therefore we limited ourselves to simulations with 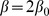 and 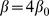 where 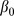 is our baseline value. Finally we also studied a system where both gap genes are activated by the same gradient (Bcd), varying both the diffusion constant 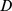 and the Kni repressor off-rate 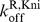, while keeping 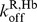 at the standard value.

### Algorithmic details

All simulations are split into a relaxation and a measurement run. During the relaxation run we propagate the system towards the steady state without data collection. To reach steady state, as a standard we run 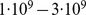 Gillespie steps (ca. 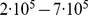 updates per nucleus). The measurement run is performed with twice the number of steps (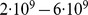). The simulations are started from exponential morphogen gradients and step profiles of the gap proteins; however, we verified that the final result was independent of the precise initial condition, and that the system reached steady state after the equilibration run. The results for 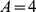 (Fig. 7) form, however, an exception: here it was impossible to obtain a reliable error bar, because of the weak pinning force on the *hb* and *kni* expression boundaries.

In steady state, we record for each row of nuclei and with a measurement interval of 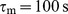 the Hb boundary position 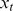, i.e. the position where 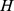 drops to half of the average steady-state value measured at its plateau close to the anterior pole, which in our simulations is equal to the maximum average total Hb level 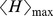. From the corresponding histogram we obtain the boundary width 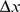 by computing the standard deviation. Additionally, after runtime we calculate an approximation for 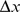 from the standard deviation of 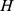 divided by the slope of the averaged 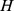 profile, both quantities taken at 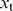, see Eq. 1 [6], [19], [35]. Further details of boundary measurement are described in Text S1.

Error bars for a given quantity are estimated from the standard deviation among 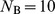 block averages (block length 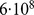) divided by 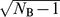, following the procedure described in [73]. We verified that estimates with smaller and larger block sizes yield similar estimates for a representative set of simulations.

## Supporting information

Text S1 **Supporting information.** More detailed information on parameter choice, data measurement and analytical estimates and results on the system with altered cooperativity, the systems with altered production dynamics and the systems where both gap genes are activated by the same morphogen gradient.
(PDF)

Video S1
**Establishment of gap gene expression patterns for a low diffusion constant of the gap proteins.** Movie of the total concentration of Hb as a function of time for 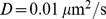 and morphogen dosage factor 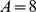, starting from zero concentration of both Hb and Kni. Note that initially small “crystallites” are formed in the overlap region where both *hb* and *kni* are activated by their respective morphogens, Bcd and Cad. These crystallites then coarsen and join the Hb domain formed near the anterior pole. The green line marks the positions where the total Hb concentration crosses the boundary threshold value.(MP4)

Video S2 **Establishment of gap gene expression patterns for a low diffusion constant of the gap proteins.** Movie of exactly the same system trajectory as in Video S1, only now showing the difference between the total Hb and total Kni copy number.
(MP4)

Video S3
**Establishment of gap gene expression patterns for a high diffusion constant of the gap proteins.** Movie of the total concentration of Hb as a function of time for 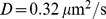 and morphogen dosage factor 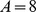, starting from zero concentration of both Hb and Kni. Note that the Hb domain emerges at the anterior pole and progresses into the overlap region. The green line marks the positions where the total Hb concentration crosses the boundary threshold value.(MP4)

Video S4 **Establishment of gap gene expression patterns for a high diffusion constant of the gap proteins.** Movie of exactly the same system trajectory as in Video S3, only now showing the difference between the total Hb and total Kni copy number.
(MP4)
