# Supplementary material for: Mutual Repression Enhances the Steepness and Precision of Gene Expression Boundaries
Source: PLoS Comput Biol. 2012 Aug 30;8(8):e1002654. doi: 10.1371/journal.pcbi.1002654 (PMC3431325; doi:10.1371/journal.pcbi.1002654)
Supplement: Text S1 — Supporting information. More detailed information on parameter choice, data measurement and analytical estimates and results on the system with altered cooperativity, the systems with altered production dynamics and the systems where both gap genes are activated by the same morphogen gradient. (PDF) [file pcbi.1002654.s001.pdf]

SUPPORTING INFORMATION
Mutual Repression enhances the Steepness and Precision of Gene Expression Boundaries

Thomas R. Sokolowski[1], Thorsten Erdmann[2], Pieter Rein ten Wolde[3,*]
1 FOM Institute AMOLF, Science Park 104, 1098 XG Amsterdam, The Netherlands
2 University of Heidelberg, Institute for Theoretical Physics, Philosophenweg 19, 69120 Heidelberg, Germany
3 FOM Institute AMOLF, Science Park 104, 1098 XG Amsterdam, The Netherlands
$*$ E-mail: tenwolde@amolf.nl

1 Methodic details

1.1 Measurement of the boundary width

By default we determine the boundary width in the following two ways:

Let $c^{\mathrm{m}}_{\mathrm{n,s}}$ be the copy number of species s in a nucleus with angular index $m < N_\phi$ and axial index $n < N_{\mathrm{x}}$, where N_ϕ is the number of rows around the circumference of the cylinder, and N_{x} is the number of colums in the axial direction along the AP axis. To compute the boundary width of the expression domain of a gap protein s, we compute for each row m $T^{\mathrm{m}}_{\mathrm{n,s}} = (c^{\mathrm{m}}_{\mathrm{n,s}} - \theta_{\mathrm{s}}) \cdot (c^{\mathrm{m}}_{\mathrm{n+1,s}} - \theta_{\mathrm{s}})$ as a function of n, where θ_{s} is half the copy number expected at full activation. A boundary position $x^{\mathrm{m}}_{\mathrm{t}} = x^{\mathrm{m}}(n_{\mathrm{t}} + \frac{1}{2})$ is defined as the position (nucleus) where $T^{\mathrm{m}}_{\mathrm{n_t,s}} < 0$. The values of $x^{\mathrm{m}}_{\mathrm{t}}$ are recorded in a histogram; here, the positions for the different rows m are put in the same histogram. The histogram is normalized at the end of the simulation, and the boundary width Δx is calculated as the standard deviation of this histogram.

Secondly, at the end of the simulation, the slope of the average, $\langle H(x_{\mathrm{t}})\rangle'$, and the standard deviation of the total Hb copy number $\sigma_{\mathrm{H}}(x_{\mathrm{t}})$ at the Hb boundary position x_{t} are calculated from the time- and ϕ-averaged profiles. From this, an approximation for the boundary width given by $\Delta x \approx \frac{\sigma(x_{\mathrm{t}})}{|\langle H(x_{\mathrm{t}})\rangle'|}$ is obtained, following [1–3]. To this end, first x_{t} is determined in the same way as in the runtime measurements, only now working on the (both time- and circumference-) averaged profile. We describe in the following section how the steepness $\langle H(x_{\mathrm{t}})\rangle'$ is measured.

1.2 Measurement of the profile steepness

In our discrete system the measurement of a local derivative at the boundary position x_{t} is a process prone to even small stochastic variations if a naive measurement technique is chosen. If the average boundary position x_{t} for a set of different samples with identical initial conditions always is in between two particular nuclear positions $x(n_0)$ and $x(n_0+1)$, then using linear differences to determine the steepness $\langle H(x_{\mathrm{t}})\rangle'$ at the boundary position may give a reasonable estimate. If, however, x_{t} fluctuates around a particular nuclear position $x(n_0)$ among different samples and $\langle H(x(n_0-1))\rangle - \langle H(x(n_0))\rangle$ significantly differs from $\langle H(x(n_0))\rangle - \langle H(x(n_0+1))\rangle$, the linear differences method will produce a large error bar and also markedly affect the mean of x_{t} among these samples. As a result both the measured steepness and the quality of that measurement for a given set of parameters depends on whether x_{t} accidently happens to predominantly vary in the interval between the same nuclear positions or not. To overcome this illness we measure the boundary steepness from the average protein profile by a two-step polynomial fitting procedure: First we fit a polynomial of 3rd degree to a region of the data around x_{t} that contains at least four points (nuclei). The derivative of the polynomial at x_{t} gives an initial estimate of the boundary slope, which we use this to calculate the approximative x-interval over which the profile falls from maximal to minimial expression level. If the latter is larger than the original fitting range (which usually is the case) we repeat the fitting on the enlarged interval. Since the profiles to a good

approximation are sigmoidal functions this improves the quality of the fit. The measured boundary slope then is defined as the derivative of the polynomial function at x_{t} after the second fitting.

1.3 Number of cortical nuclei at cell cycle 14

The development of the Drosophila embryonic syncytium starts with a single nucleus. The first 9 nuclear divisions happen in the yolk. During cell cycles 7 to 10 a migration of the nuclei towards the cortex can be observed. However, approximately 200 polyploid nuclei stay behind in the yolk and stop dividing after their 10th cycle [4]. This quiescence persists during subsequent cell cycles, including cycle 14. As an effect of this, the number of nuclei at the cortex in cycle 14 is considerably lower than $2^{13} = 8192$. An estimate of the reduced number of cortical nuclei is given by:

$$N_{\mathrm{cortex}} \simeq (2^9 - 200) \cdot 2^4 = 4992 \tag{1}$$

This number indeed is closer to 2^{12} than to 2^{13}. Note that in our model the precise number of nuclei does not matter, rather it is the distance between the nuclear compartments and the diffusion correlation length that impact on the results. Our values for both the internuclear distance and the nuclear diameter correspond to the experimental values reported by Gregor et al. [2, 5].

1.4 Predicted copy numbers and effective protein lifetime

Our main observables are the total copy numbers of Hb and Kni, defined as follows:

$$\begin{aligned} H \equiv c^{\mathrm{m}}_{\mathrm{n,H}} &= c^{\mathrm{m}}_{\mathrm{n,H_M}} + 2c^{\mathrm{m}}_{\mathrm{n,H_D}} + 2\sum_{\mathrm{j=0}}^{5} c^{\mathrm{m}}_{\mathrm{n,K^1_j}} \\ K \equiv c^{\mathrm{m}}_{\mathrm{n,K}} &= c^{\mathrm{m}}_{\mathrm{n,K_M}} + 2c^{\mathrm{m}}_{\mathrm{n,K_D}} + 2\sum_{\mathrm{j=0}}^{5} c^{\mathrm{m}}_{\mathrm{n,H^1_j}} \end{aligned} \tag{2}$$

Here, for $G \in \{H, K\}$, $c^{\mathrm{m}}_{\mathrm{n,G^1_j}} = 1$ if the promoter of species G is binding j morphogen molecules and one (repressing) gap dimer; evidently, at any given moment in time $c^{\mathrm{m}}_{\mathrm{n,G^1_j}}$ can be equal to one for only one $j \in \{0..5\}$.

The ratio between the number of monomeric and the number of dimeric proteins is a nontrivial function of the monomer production rate, the monomer and dimer degradation rates and the parameters that determine the dimerization and dedimerization reactions. To obtain an estimate for the expected copy numbers of monomers and dimers of gene g we solved the mean-field rate equations for a simplified model which comprises monomer production, (de)dimerization and monomer and dimer degradation only, i.e. in which promoter state fluctuations and diffusion are neglected, in the steady state. We assume here that stochastic monomer production events can be accounted for by an effective mean-field production rate $\langle\beta\rangle = \beta\langle H^0_5\rangle$ for Hb and similarly for Kni, which depends on promoter (un)binding parameters and the particular morphogen and repressor levels. This yields the following prediction for the copy number

of monomers ($G_{\mathrm{M},\langle\beta\rangle}$) and dimers ($G_{\mathrm{D},\langle\beta\rangle}$):

$$\begin{aligned} G_{\mathrm{M},\langle\beta\rangle} &= \frac{1}{4k_{\mathrm{on}}^{\mathrm{D}}\mu_{\mathrm{D}}}\Bigg\{2k_{\mathrm{on}}^{\mathrm{D}}\mu_{\mathrm{D}} - k_{\mathrm{off}}^{\mathrm{D}}\mu_{\mathrm{M}} - \mu_{\mathrm{M}}\mu_{\mathrm{D}} \\ &\qquad + \sqrt{8\langle\beta\rangle k_{\mathrm{on}}^{\mathrm{D}}\mu_{\mathrm{D}}\left(k_{\mathrm{off}}^{\mathrm{D}} + \mu_{\mathrm{D}}\right) + \left[\mu_{\mathrm{M}}\left(k_{\mathrm{off}}^{\mathrm{D}} + \mu_{\mathrm{D}}\right) - 2k_{\mathrm{on}}^{\mathrm{D}}\mu_{\mathrm{D}}\right]^2}\Bigg\} \\ G_{\mathrm{D},\langle\beta\rangle} &= \frac{1}{8k_{\mathrm{on}}^{\mathrm{D}}\mu_{\mathrm{D}}^2}\Bigg\{k_{\mathrm{off}}^{\mathrm{D}}\mu_{\mathrm{M}}^2 + \mu_{\mathrm{D}}\left[4\langle\beta\rangle k_{\mathrm{on}}^{\mathrm{D}} + \mu_{\mathrm{M}}\left(\mu_{\mathrm{M}} - 2k_{\mathrm{on}}^{\mathrm{D}}\right)\right] \\ &\qquad - \mu_{\mathrm{M}}\sqrt{8\langle\beta\rangle k_{\mathrm{on}}^{\mathrm{D}}\mu_{\mathrm{D}}\left(k_{\mathrm{off}}^{\mathrm{D}} + \mu_{\mathrm{D}}\right) + \left[\mu_{\mathrm{M}}\left(k_{\mathrm{off}}^{\mathrm{D}} + \mu_{\mathrm{D}}\right) - 2k_{\mathrm{on}}^{\mathrm{D}}\mu_{\mathrm{D}}\right]^2}\Bigg\} \end{aligned} \tag{3}$$

Here μ_{M} (μ_{D}) is the monomeric (dimeric) degradation rate and $k_{\mathrm{on}}^{\mathrm{D}}$ (k_{off}^{D}) are the dimerization forward (backward) rates, respectively. From this we calculate the total expected copy number $G_{\langle\beta\rangle} := 2G_{\mathrm{D},\langle\beta\rangle} + G_{\mathrm{M},\langle\beta\rangle}$ at effective production rate $\langle\beta\rangle$. In particular in the full-activation case, i.e. when the probability to be fully activated and unrepressed $\langle G_5^0\rangle \approx 1$ and therefore $\langle\beta\rangle \approx \beta$, the above estimates correspond to average values from our simulations very well.

We define the effective degradation as $\mu_{\mathrm{eff}} = \mu_{\mathrm{eff}}(G_{\mathrm{M}}, G_{\mathrm{D}}) = \frac{1}{G_{\mathrm{M}}+2G_{\mathrm{D}}}\left(\mu_{\mathrm{M}}G_{\mathrm{M}} + 2\mu_{\mathrm{D}}G_{\mathrm{D}}\right)$. Our standard values result in $\mu_{\mathrm{eff}} \approx 4.34 \cdot 10^{-3}$ /s with $G_{\mathrm{M}} = G_{\mathrm{M},\beta}$ and $G_{\mathrm{D}} = G_{\mathrm{D},\beta}$.

2 Additional analysis

2.1 Poissonian limit with dimerization

In [3], it was shown that, when $D \to \infty$, the variance in the protein concentration becomes equal to the mean concentration: diffusion washes out bursts in gene expression, thus reducing the non-Poissonian part of the noise. However, in that model the proteins do not dimerize, in contrast to our model. With dimerization, a different limit for the variance in the total protein concentration is approached as $D \to \infty$. To derive this limit, first note that the total protein copy number G of a protein G is $G \approx 2G_{\mathrm{D}} + G_{\mathrm{M}}$. Assuming that $\langle G_{\mathrm{D}}G_{\mathrm{M}}\rangle \approx \langle G_{\mathrm{D}}\rangle\langle G_{\mathrm{M}}\rangle$ (our simulations indicate that this approximation is very accurate), we find that the variance σ_{G}^2 in G is:

$$\sigma_{\mathrm{G}}^2 \approx 4\sigma_{\mathrm{G_D}}^2 + \sigma_{\mathrm{G_M}}^2, \tag{4}$$

where $\sigma_{\mathrm{G_D}}^2$ is the variance in the dimer level G_{D} and $\sigma_{\mathrm{G_M}}^2$ is the variance in the monomer level G_{M}. Both monomers and dimers are subject to spatial averaging, and therefore their variances can be written in the form [3]:

$$\begin{aligned} \sigma_{\mathrm{G_M}}^2 &= G_{\mathrm{M}} + \frac{1}{N}\left(\sigma_{0,\mathrm{G_M}}^2 - G_{\mathrm{M}}\right) \\ \sigma_{\mathrm{G_D}}^2 &= G_{\mathrm{D}} + \frac{1}{N}\left(\sigma_{0,\mathrm{G_D}}^2 - G_{\mathrm{D}}\right) \end{aligned} \tag{5}$$

Here N is the number of nuclei contributing to the averaging, which is proportional to D, and $\sigma_{0,\mathrm{G_{M/D}}}^2$ is the variance in the monomer and dimer levels in the absence of diffusion, respectively. The part preceded by $1/N$ represents the variance that can be reduced by spatial averaging. Plugging these expressions into

the previous and using $G = 2G_{\mathrm{D}} + G_{\mathrm{M}}$ we arrive at:

$$\begin{aligned}
\sigma_{\mathrm{G}}^2 &= 4G_{\mathrm{D}} + G_{\mathrm{M}} + \frac{1}{N}\left[4\sigma_{0,\mathrm{G_D}}^2 + \sigma_{0,\mathrm{G_M}}^2 - 4G_{\mathrm{D}} - G_{\mathrm{M}}\right] \\
&= \left(1 + \frac{2G_{\mathrm{D}}}{G}\right)G + \frac{1}{N}\left[\sigma_{0,\mathrm{G}}^2 - \left(1 + \frac{2G_{\mathrm{D}}}{G}\right)G\right] \\
&=: \left(1 + f_{\mathrm{D}}\right)G + \frac{1}{N}\left[\sigma_{0,\mathrm{G}}^2 - \left(1 + f_{\mathrm{D}}\right)G\right] \qquad (6)
\end{aligned}$$

Note that N is the same for both monomers and dimers because their diffusion constant does not differ in our model. Evidently, the lower bound for σ_{G} in the limit $N \to \infty$ is not $\sqrt{G}$ any more, but given by $\sqrt{(1 + f_{\mathrm{D}})\,G}$, where f_{D} is the fraction of proteins in the dimer state with respect to the total protein number (implying $f_{\mathrm{D}} \leq 1$). This is indeed what we observe in our data for σ_{G}. In our simulations the equilibrium is strongly shifted towards the dimerized state, so that $f_{\mathrm{D}} \approx 0.97$. We can understand the limit $N, D \to \infty$ intuitively by noting that in this limit there is no noise in the nuclear protein concentration due to the stochastic production and decay of molecules in each of the nuclei—this is because the synthesized molecules are immediately donated to a reservoir that is infinitely large; instead, there is only noise in the nuclear protein concentration due to the sampling of molecules from this reservoir, which obeys Poissonian statistics: $\sigma_{\mathrm{G_M}}^2 = G_{\mathrm{M}}$ and $\sigma_{\mathrm{G_D}}^2 = G_{\mathrm{D}}$. This yields, for $N, D \to \infty$, $\sigma_{\mathrm{G}}^2 = 4\sigma_{\mathrm{G_D}}^2 + \sigma_{\mathrm{G_M}}^2 = 4G_{\mathrm{D}} + G_{\mathrm{M}} = (1 + f_{\mathrm{D}})G$.

2.2 Bifurcation analysis

In order to predict the regions in which bistability can be expected for different amplitudes A of the morphogen gradients we performed a deterministic mean-field bifurcation analysis for a simplified 1-dimensional version of our model of mutual repression between *hb* and *kni*. The analysis is based on the following two equations describing the change of the mean-field total copy number of Hb ($H(x)$) and Kni ($K(x)$) at position x:

$$\partial_{\mathrm{t}} H(x) = \beta_{\mathrm{H}}(x)\frac{K_{\mathrm{R}}^2}{K_{\mathrm{R}}^2 + [f_{\mathrm{D}}K(x)]^2} - \mu_{\mathrm{H}}H(x) \qquad (7)$$

$$\partial_{\mathrm{t}} K(x) = \beta_{\mathrm{K}}(x)\frac{K_{\mathrm{R}}^2}{K_{\mathrm{R}}^2 + [f_{\mathrm{D}}H(x)]^2} - \mu_{\mathrm{K}}K(x) \qquad (8)$$

Here β_{H} and β_{K} represent the protein synthesis rates, μ_{H} and μ_{K} the corresponding (effective) degradation rates, K_{R} is the dissociation constant of cooperative repressor binding to the promoter and f_{D} is the fraction of proteins in the dimerized state. Note that, since the intermediate step of dimerization is neglected here, we have to take $K_{\mathrm{R}} = \sqrt{k_{\mathrm{off}}^R / k_{\mathrm{on}}^R}$ if k_{on}^R and k_{off}^R are the binding rates of the dimers. To facilitate calculations we make two further simplifying assumptions here:

1. We neglect activation dynamics and resulting promoter state fluctuations, i.e. we assume that certain constant levels of the activators at position x lead to average constant production rates $\beta_{\mathrm{H}} = \beta([Bcd](x))$ and $\beta_{\mathrm{K}} = \beta([Cad](x))$, respectively. In our standard case $\beta([Act](x)) = [Act]^5(x)/([Act]^5(x) + 690^5)$ for both $[Act] = [Bcd]$ and $[Act] = [Cad]$.

2. In our simulations we have different degradation rates for monomers and dimers so that the effective total degradation rate depends on the monomer-to-dimer ratio, which in turn varies with the total copy number (see section 1.4). Thus, in principle, also f_{D} and μ_{H} and μ_{K} are functions of x, or the corresponding activator levels. Since this introduces further nonlinearities into the above equations and complicates their solution, we substitute the degradation rates μ_{H} and μ_{K} by a

constant value μ_{eff}, which is the effective degradation rate for the maximal expression level (full activation). Also for f_{D} we take the constant value for full activation, $f_{\text{D}} \simeq 0.97$, which reflects that the dimerization equilibrium in our simulations is strongly shifted towards the dimerized state. The predictions concerning the bifurcation behavior only change marginally if μ_{eff} and f_{D} values for lower expression levels are used.

For each position x with local activator levels corresponding to the ones in the simulations we calculated fixed point solutions for the copy number pair $(H(x), K(x))$ starting from the steady-state assumption $\partial_{\text{t}}(H(x), K(x)) = (0, 0)$. The stability of the fixed points was determined starting from the Jacobian for the above ODE system:

$$J(H, K) = \begin{pmatrix} \partial_{\text{H}}\left[\partial_{\text{t}} H\right] & \partial_{\text{K}}\left[\partial_{\text{t}} H\right] \\ \partial_{\text{H}}\left[\partial_{\text{t}} K\right] & \partial_{\text{K}}\left[\partial_{\text{t}} K\right] \end{pmatrix} \tag{9}$$

Within the relevant parameter regime we obtained fixed points with either two negative eigenvalues (i.e. stable fixed points) or one positive and one negative eigenvalue (i.e. saddle points). The determinant therefore completely characterizes the stability of the fixed points. If $\det J(H_0, K_0) < 0$, then (H_0, K_0) is a saddle point. Otherwise it is stable.

Fig. S1 shows the fixed point solutions for Hb and Kni as a function of x for different activator amplitudes A. Stable solutions are drawn with solid, unstable solutions with dashed lines. Depending on the A value, the system displays a saddle node bifurcation at a point towards the anterior (Hb) or posterior (Kni) from midembryo. Within the region confined by the bifurcation points two stable and one unstable fixed points exist for each gene, implying bistability. The region clearly widens for increasing A and spans almost the whole embryo length for $A = 8$. Our deterministic analysis therefore predicts the enlargement of the region of bistability as observed in our single nucleus simulations.

2.3 Estimation of switching times

To quantify the swiching times in the presence of bistability we performed simulations of isolated single nuclei featuring the same set of reactions and parameters as in the full scale simulation. To obtain estimates of switching times at different positions x along the AP axis we set the levels of Bcd and Cad in the given nucleus equal to the ones at x in the space-resolved simulations. The switching time was estimated by calculating from long time trajectories of the total Hb and Kni copy numbers the relaxation time t_{s} of the average correlation function

$$\langle C(t) \rangle_{\text{t}_0} \equiv \frac{\langle I_{\text{H}}(t_0) I_{\text{K}}(t) \rangle_{\text{t}_0}}{\langle I_{\text{H}}(t_0) \rangle_{\text{t}_0}} \tag{10}$$

where I_{H} (I_{K}) are indicator functions which are one if the difference in the total gap gene copy numbers $\Delta N = H - K$ is above (below) a certain threshold Θ_{N} ($-\Theta_{\text{N}}$). Θ_{N} thus defines the regions within which the switch is considered to have switched to the Hb–high or Kni–high states, respectively, and serves to separate the stable attractor states from the transition region. We found that $\Theta_{\text{N}} = 200$ is a reasonable choice for our set of parameters.

We determined the switching times from one long sample for different positions x and different activator amplitudes A and find that t_{s} is very similar within the double-activated bistable regions for high A. To obtain an error estimate we additionally calculated block averages of estimated switching times among 10 long samples for various A at midembryo ($x = L/2$). Table S1 shows our results from the latter procedure.

Note that for $A = 1$ the system is not truly bistable yet because for $A = 1$ we have half-activation at midembryo and due to the lack of diffusion large promoter-state fluctuations dominate over long-time switching potentially induced by mutual repression. Consequently, the given number does not reflect a switching time. We cite it here for completeness, however.

Table S1. Switching times at midembryo for different activator levels.

Activator amplitude A	Switching time $t_s[s]$
(1)	(6343.7 ± 17.2)
2	20302.5 ± 74.5
4	20957.3 ± 54.9
8	20994.7 ± 67.2

2.4 Analysis of statistical properties of the boundary

Our measures for both the boundary steepness and the variance of the boundary position are based on averages over both the time and the circumference of the embryo which were calculated during runtime. While the double-averaging procedure limits the amount of data that must be stored and facilitates rapid acquisition of good statistics, it also discards information about the microscopic properties of the boundary at a given time instance. Based on the average data it is impossible to determine whether the blurring of the boundary quantified by Δx is due to concerted stochastic movements of a steep and rather homogeneous instantaneous boundary or simply due to stochastic fluctuations of the boundary position in each nuclear row around a well-defined constant mean boundary position (or due to both). In the latter case the boundary will be rough at each given time instance, i.e. the time average of the boundary position variance in the cicumferential direction will be large, but the time variance of its circumferential mean will be negligible. The opposite will be the case in the other extreme. These quantities therefore can be used to distinguish the two hypothetical situations. The overall boundary width in both cases is given by the sum:

$$\Delta x^2 = \overline{\sigma^2_{\mathrm{x_t}(\phi)}} + \sigma^2_{\langle \mathrm{x_t} \rangle_\phi (\mathrm{t})} \tag{11}$$

Here $\langle \dots \rangle_\phi$ denotes the average over the circumference, while the bar denotes the time average. An identical variance decomposition can be made for the fluctuations of the Hb copy number at any position x along the AP axis. Similarly, comparing the average of the profile steepness for a particular nuclear row and time instance to the steepness of the time- and circumference average of the copy number reveals whether the steepness of the average profile is due to concerted movements of similarly shallow instantaneous profiles or due to unconcerted fluctuations of steep instantaneous profiles.

In order to determine which of the portrayed blurring mechanisms is dominant in our system we performed the described variance decomposition for a set of 100 instantaneous outputs of the fully resolved 2D system in steady state, i.e. for 6400 different total Hb copy number profiles along the AP axis, for both the variances at the boundary and for the steepness at the boundary and for both the system with and without mutual repression. We focused on our standard parameter set (see Table S2) and a range of gap protein diffusion constants D.

2.4.1 At each time instance the boundary is rather rough

Fig. S2 shows for the systems with (Fig. S2A and C) and without (Fig. S2B and D) mutual repression the variance decomposition for the variance of the Hb copy number at the boundary (Fig. S2A and B) and for the variance of the boundary position x_t (Fig. S2C and D) as a function of the Hb protein diffusion constant D. As a control we compare the total variances calculated from the instantaneous profiles to the variances accumulated during runtime and, in case of Δx, to the value obtained from the approximation $\Delta x = \sigma_{\mathrm{H}}(x_t)/|\langle H(x_t)\rangle'|$ (note that here $\langle \dots \rangle$ is the average over both time and ϕ). We see a good agreement between these quantities. The plots reveal that both for $\sigma_{\mathrm{H}}(x_t)$ and Δx the variance over the circumference at a fixed time is by far the dominant contribution to the overall variance. This implies that in our system the boundary is indeed very rough at each time point and that concerted boundary movements do not occur.

2.4.2 At each time instance the profiles are slightly steeper than their average

The calculation of the variance decomposition is less straightforward for the slope. In particular for low D, when spatial averaging is still inefficient, the instantaneous profiles are very ragged and the boundary threshold value typically is crossed at multiple positions along the AP axis. This makes it impossible to uniquely define an instantaneous boundary position as required to calculate the instantaneous boundary slope. In order to perform the analysis at least on a subset of the data we introduced a protocol which only takes into account instantaneous profiles with a single boundary crossing, rejecting all other profiles. For low D, however, the rejection rates rise above 90%. We therefore decided to smoothen the profiles by computing running averages between a fixed number ν of nuclei along the AP axis before the analysis. The averaging lowers the rejection rate dramatically, however it also decreases the profile steepness and therefore manipulates the observable of interest. Nevertheless we can make a qualitative statement on the base of the results obtained for only slight smoothening of the profiles ($\nu = 3$). For simplicity and due to increased data abundance, in this analysis we used simple finite differences to determine the slope.

In Fig. S3 we plot the average of the instantaneous boundary steepness for different degrees of smoothening (averaging over $\nu = 3, 5, 7$ nuclei along the x-axis) as a function of D and compare to the steepness of the average Hb profile for the system with (S3A) and without (S3B) mutual repression. While the data for $\nu > 3$ clearly must be considered biased by the running averages, the values for $\nu = 3$ show that the instantaneous boundary slope on average is higher than the slope of the average profile, in particular for low diffusion constants.

The variance decomposition for the boundary position x_{t} shows that the variance of the circumference mean of the boundary position in time is very small. This implies that the steepness of the circumference-averaged profiles should be approximately equal to the steepness of the time- and circumference-averaged profile. As a control we therefore repeated the above analysis on the 100 ϕ-averaged instantaneous profiles of the same dataset. The averaging along the circumference significantly reduces the number of profiles with ambiguous boundary positions. We therefore were able to obtain reasonable estimates of the observable without pre-smoothening of the profiles ($\nu = 1$). The results are shown in Fig. S3C for the system with mutual repression and Fig. S3D for the system without mutual repression. In the system with mutual repression the average slope of the ϕ-averaged profiles for $\nu = 1$ agrees well with the slope of the both time- and ϕ-averaged Hb profile. In the system without mutual repression the ϕ-averaged profiles are slightly steeper than the average.

3 Additional simulations

3.1 Influence of the Hill coefficient

To address the influence of changing activator cooperativity on our results we performed simulations with reduced number of activator binding sites n_{max}. While in our model this is achieved by simply reducing the number of intermediate states between the empty promoter state and the producing promoter state, the binding parameters have to be rescaled with care to preserve the activation equilibrium at midembryo. Since we assume the activator binding rates to be diffusion limited, the necessary changes affect the unbinding rates $k^{\mathrm{A}}_{\mathrm{off,n}} = a/b^n$. However, even when preserving the equilibrium, the freedom in the choice of these parameters allows for altering the time scale of transitions between the different activation levels. In order to rescale the rates in a unique fashion upon lowering n_{max} we imposed the following constraints:

1. For all n_{max} the effective activator dissociation constant at midembryo $K^{\mathrm{A}}_{\mathrm{D}} = A_{1/2} = 690$ is preserved, which implies that for all n_{max} the average activation probability at midembryo is 1/2.

2. The waiting-time distribution for the unbinding from the producing state is the same for all n_{max} and, for comparison, equal to the one for the default cooperativity $n_{max} = 5$, i.e. $\forall n : k^{\mathrm{A}}_{\mathrm{off,n_{max}}} = const = k^{\mathrm{A}}_{\mathrm{off,5}}$.

3. The off-rate reduction per subsequent activator binding is always $1/b$, i.e. $\forall n : b(n) = b$.

Note that for $n_{max} = 1$ the first two conditions can be met together only if $K_{\rm D}^{\rm A} k_{\rm on}^{\rm A} = k_{\rm off,5}^{\rm A}$, which is not the case for our parameter set. We therefore restricted ourselves to $n_{max} \in \{2, 3, 4, 5\}$. For each n_{max}, the above constraints were used to uniquely determine the parameters a and b from the exact analytical solution for the average occupancy of the producing state, which was obtained from steady-state mean-field solutions of the chemical mass-action ODEs. Interestingly this results in only minor differences in a among the different n_{max} values, while the reduction per binding step $1/b_{\rm n_{max}}$ becomes significantly larger for lower n_{max}. This fact has an important implication for the noise charactistics of the different promoters: If $a_{\rm n_{max}} \simeq a = const$ for all n_{max} then the unbinding rate from the state binding $(n_{max} - 1)$ activator proteins (the "highest" non-producing activator state) is given by:

$$k_{\rm off,(n_{max}-1)}^{\rm A} \simeq a/b_{\rm n_{max}}^{(n_{max}-1)} = b_{\rm n_{max}} k_{\rm off,5}^{\rm A}$$

Since $b_{\rm n_{max}}$ markedly increases with decreasing Hill coefficient the off-rate $k_{\rm off,(n_{max}-1)}^{\rm A}$ for low n_{max} will be higher than the corresponding rate for high n_{max}. This will favor rapid returns to the producing state with n_{max} bound activator molecules for high Hill coefficients, whereas for low Hill coefficients the promoter is more likely to descent into the regime with less activator molecules bound. The fact that this is less likely for higher Hill coefficients is compensated by the fact that also the time to return to the producing state from the states binding low numbers of activator molecules on average is longer for higher n_{max}. Note that the mean off-time–just as the mean on-time–is the same for all n_{max}. In short, for the promoters with higher Hill coefficients we expect an off-time distribution with high probability weight on short off-times and a long low-probability tail for long off-times, while the distribution for lower Hill coefficients should resemble an exponential.

In order to illustrate this effect we recorded long time-trajectories of the occupancy of the producing state in a single isolated nucleus close to midembryo for different n_{max} and without mutual repression nor diffusion. All other parameters were kept at the standard values. From these trajectories we determined the on- and off-times of the promoter and binned them into a histogram. The results are shown in Fig. S5. It can be seen that while for $n_{max} = 2$ the two distributions are exponential with approximately equal mean, the off-times distribution increasingly deviates from an exponential distribution as n_{max} is increased; more probability is shifted to very short off-times and very long off-times, causing the emergence of a long tail in the distribution.

3.1.1 Also for lower Hill coefficients mutual repression steepens profiles without corrupting boundary precision

The broadening of the off-times distribution is expected to result in higher output noise for high n_{max} as compared to low n_{max}. This is confirmed by the simulations of the full-scale spatially resolved system for different n_{max}. Fig. S6 shows $\sigma_{\rm H}(x_{\rm t})$, the average standard deviation of the total Hb copy number at the boundary position $x_{\rm t}$ (upper panels), the steepness $|\langle H(x_{\rm t})\rangle'|$ of the average Hb profile at $x_{\rm t}$ (middle panels) and the boundary width Δx (lower panels) as a function of the gap protein diffusion constant D for $n_{max} \in \{2, 3, 4, 5\}$. $\sigma_{\rm H}(x_{\rm t})$ is indeed decreasing upon lowering n_{max}, in particular in the regime of low diffusion constants. For higher diffusion constants the decrease is less pronounced: spatial averaging is efficient enough to lower the output noise down to the observed values irrespective of the width of the off-time distribution. The noise decreases less markedly for the systems with mutual repression. This is most likely due to the fact that lowering n_{max} also increases the probability of occasional repressor production beyond midembryo, which in turn increases the noise. The steepness plots reveal that, although the profiles naturally become less steep for lower n_{max}, the steepness in the systems with mutual repression is markedly higher than the one in the system without mutual repression. In all systems the steepness as a function of D shows a very similar behavior: Upon increasing D the steepness in the systems with mutual repression first increases towards a maximum before it rapidly decreases. Since both $\sigma_{\rm H}(x_{\rm t})$ and

$|\langle H(x_t)\rangle'|$ change with n_{max} in a similar fashion, in particular in the region around $D = 1$ μm^2/s, the width Δx as a function of D also looks very similar in this region for all n_{max}. In all cases the profiles in the system with mutual repression are more precise and markedly steeper as compared to the system without mutual repression at a D-value which is one order of magnitude less than the optimal value in the systems without mutual repression. Therefore the basic effect observed in our simulations for $n_{max} = 5$ persists in the simulations for lower Hill coefficients.

3.1.2 Lower Hill coefficients allow for stronger morphogen level variations

Although lowering n_{max} in our system reduces the protein production noise it also markedly decreases the steepness of the gene activation profiles. An important implication of this is that for lower n_{max} the activation probability beyond midembryo increases. Lowering n_{max} thus is similar to increasing the activator amplitude A and, in principle, might result in the creation of a bistable region around midembryo already for lower A-values as compared to the system with $n_{max} = 5$. We analysed how the results for Δx as a function of A for the case of correlated variations change as n_{max} is decreased. Fig. S7 shows $\Delta x(A)$ for $n_{max} \in \{2, 3, 4, 5\}$ and $D = 1.0$ μm^2/s for systems with and without mutual repression. Overall, $\Delta x(A)$ is very similar for all considered n_{max}. For $A \leq 2$ the width Δx in the systems with mutual repression is always lower than in the systems without mutual repression. The minimal Δx is attained at $A = 1$ in all cases. The main difference is in how Δx changes with A for $A > 1$: The lower n_{max}, the slower the width increases with A. Thus, while lower Hill coefficients decrease the steepness of the profiles significantly, they may prove beneficial by extending the range over which extrinsic variations are successfully buffered without increasing intrinsic fluctuations of the boundary.

3.2 Influence of the expression level

In order to examine the influence of a changed signal-to-noise ratio on our results we performed simulations with altered production dynamics. We did this by (1) introducing bursty production, i.e. producing 10 copies of the gap protein monomer at a time with a 10 times lower production rate ($\beta = \beta_0/10$), and (2) by keeping the burst size at one and changing the production rate β. To preserve the binding equilibrium of the repression reaction at midembryo upon changing β we also changed the off-rate of the repressor dimers by a factor f_β^{D}, which is the ratio between the expected number of dimers at midembryo for the altered production rate β and the corresponding value for the standard production rate β_0. Note that, since in our system the copy numbers of both monomers and dimers depend on β in a nontrivial fashion (see section 1.4) the effective copy number increase typically does not correspond to the ratio β/β_0. Therefore $f_\beta^{\mathrm{D}} > \beta/\beta_0$ for $\beta > \beta_0$.

3.2.1 Bursty production has only a marginal influence on the boundary properties

In Fig. S8 we plot the standard deviation of the total Hb copy number at the boundary, the steepness of the total Hb copy number profile at the boundary and the boundary width Δx as a function of D for the system with bursty production (burst size 10). There is no significant difference as compared to the system with normal production (burst size 1, compare to Fig. S6(D) or Fig. 3 of main article). For low D the production noise is marginally higher with bursty production, resulting in a slight increase of Δx in this regime; the effect of varying D, however, is much more important. This is most likely a consequence of the fact that for the given Hill coefficient $n_{max} = 5$ promoter state fluctuations are already at a high level due to a very broad off-time distribution (see section 3.1).

3.2.2 Increased production rates reveal different noise scaling behavior for different regimes of the diffusion constant

Upon increasing the production rate and consequently the total copy number of the gap proteins we may expect a relative decrease in the output noise, but only if the latter is purely Poissonian. In our system this corresponds to the limit of high gap protein diffusion constants. In that limit, we expect $\sigma_{\rm G} \propto \sqrt{G}$, where $\sigma_{\rm G}$ is the noise in the total gap gene copy number G. However, in the abscence of spatial averaging, i.e. for the limit $D \rightarrow 0$, non-Poissonian noise prevails and the expected scaling is $\sigma_{\rm G} \propto G$ [3]. If the copy number profile is scaled uniformly at each AP position x, which–to a good approximation–is the case in our system, we expect for the scaling of the gradient at midembryo $G'(x_{\rm t}) \propto G(x_{\rm t})$. The expected scaling for the boundary width Δx then is $\Delta x \propto 1$ for low diffusion constants and $\Delta x \propto 1/\sqrt{G}$ for high D. While the overall characteristics of the boundary are very similar to the system with $\beta = \beta_0$, a comparison roughly confirms the predicted scaling. Fig. S9 compares for Hb the standard deviation of the total copy number at the boundary (S9A), the steepness at the boundary (S9B) and the resulting boundary width (S9C) as a function of D for increased production rates $\beta = 2\beta_0$ and $\beta = 4\beta_0$ to the corresponding values for the sytem with production rate $\beta/2$. Thus, the values for $\beta = 4\beta_0$ are compared to $\beta = 2\beta_0$ and the values for $\beta = 2\beta_0$ are compared to β_0. Blue lines mark the expected change of the quantities as predicted by the scaling relations, where the corresponding copy number increase is given by the factor $f_2 \equiv f_{2\beta_0} = 2.22$ and $f_4 \equiv f_{4\beta_0}/f_{2\beta_0} = 2.16$. Here $f_\beta \simeq f_\beta^{\rm D}$ is the predicted *total* copy number at midembryo divided by the corresponding value for $\beta = \beta_0$.

The plots show that while the slope ratio is roughly equal to f_2 (f_4) for all D both in the system with (green) and without (red) mutual repression, the noise ratio depends on the diffusion constant and also slightly differs for the systems with and without mutual repression. Nevertheless the predicted scaling behavior is confirmed in both cases: in the low diffusion constant regime the noise ratio is roughly f_2 (f_4) and approaches $\sqrt{f_2}$ ($\sqrt{f_4}$) as D increases; together this leads to a boundary width ratio of one for low D which decreases towards $1/\sqrt{f_2}$ ($1/\sqrt{f_4}$) for higher D.

3.3 Activation of both gap genes by a single gradient

In the one-morphogen gradient scenario, both *hb* and *kni* are activated by the Bcd gradient. Here, *kni* is activated in the same way as *hb*, namely by 5-step cooperative binding, but with a lower activation threshold. This results in the induction of both genes in the anterior half of the embryo up to the posterior Hb boundary and of *kni* in an additional region posterior to the Hb boundary. Given that *hb* represses *kni* more strongly than vice versa in the double-activated bistable region, this parameter choice will result in the formation of two neighboring domains. We chose the *kni* activation threshold to be lower by a factor of 1/2, which causes an offset of its half-activation point by approximately 10 nuclei (83 μm) towards the posterior. We varied the protein diffusion coefficient D and $k_{\rm off}^{\rm R,K}$, the off-rate of the Kni repressor dimers from the *hb* promoter. The rate for dissociation of the Hb dimers from the *kni* promoter was kept at the standard value $k_{\rm off}^{\rm R,H} = 5.27 \cdot 10^{-3}$ /s in all simulations.

The diffusion constant D of the gap proteins and the dissociation rate $k_{\rm off}^{\rm R,K}$ of Kni from the *hb* promoter are indeed key parameters. On the one hand, *hb* must repress *kni* more strongly than the other way around, because otherwise there will be only one *kni* domain. On the other hand, when $k_{\rm off}^{\rm R,K}$ is high, then *kni* is only significantly expressed when D is low, because *kni* represses *hb* more weakly than vice versa, which means that low amounts of invading Hb dimers are sufficient to shut off Kni production almost completely; indeed, in this regime, *kni* has hardly any effect on the precision of the *hb* expression domain. We found that when $k_{\rm off}^{\rm R,H}/k_{\rm off}^{\rm R,K}$ is roughly between 0.1 and 1, both *hb* and *kni* domains are formed robustly. In Fig. S4 we display the case for $k_{\rm off}^{\rm R,H}/k_{\rm off}^{\rm R,K} = 1/\sqrt{10} \approx 1/3$.

Fig. S4A shows that the maximum of the average Kni copy number is lower than that of Hb, even though for $x < 60$ %EL *kni* is essentially fully activated by Bcd (Fig. S4B). The lower maximum is due to the fact that *hb* represses *kni* more strongly than vice versa. Another point worthy of note is that the

fluctuations in the Kni copy number in the Kni domain are higher than those of Hb in the Hb domain (Fig. S4C). This is essentially due to the small width of the Kni domain: *kni* is either fully activated by Bcd yet still repressed by Hb or not repressed by Hb yet stochastically activated by Bcd.

Panels D-F show, respectively, the noise in the Hb copy number at the *hb* expression boundary, the steepness of this boundary, and the width of this boundary, as a function of the diffusion constant D of the gap proteins. It is seen that the results are highly similar to those of the two-gradient motif. The noise in the Hb copy number at the boundary is not much affected by mutual repression (panel D), while the steepness, and consequently boundary precision, is markedly enhanced by mutual repression, especially when the diffusion constant is small. Note that while for the two-morphogen gradient scenario the approximation $\Delta x \approx \sigma_{\rm H}(x_{\rm t})/|\langle H(x_{\rm t})\rangle'|$ is in very reasonable agreement with Δx as measured from the distribution of threshold crossings $p(x)$, here the agreement is much less. This is due to sporadic repression events in the anterior region where *hb* and *kni* are both fully activated, which leads to a long tail of $p(x)$ extending towards the anterior pole; while $p(x)$ in the tail is small, the fact that the tail is long does markedly increase the standard deviation Δx. Given that the approximation $\Delta x \approx \sigma_{\rm H}(x_{\rm t})/|\langle H(x_{\rm t})\rangle'|$ works so well for all the other cases, we consider this approximation, which does not suffer from sporadic but strong *hb* repression events in the anterior, to be more reliable. We therefore conclude that also in the one-morphogen gradient scenario, mutual repression can enhance both the steepness and the precision of gene-expression boundaries.

References

1. Tostevin F, ten Wolde PR, Howard M (2007) Fundamental limits to position determination by concentration gradients. PLoS Comput Biol 3: e78.

2. Gregor T, Tank DW, Wieschaus EF, Bialek W (2007) Probing the limits to positional information. Cell 130: 153–164.

3. Erdmann T, Howard M, ten Wolde PR (2009) Role of spatial averaging in the precision of gene expression patterns. Phys Rev Lett 103: 258101.

4. Foe VE, Alberts BM (1983) Studies of nuclear and cytoplasmic behaviour during the five mitotic cycles that precede gastrulation in drosophila embryogenesis. J Cell Sci 61: 31–70.

5. Gregor T, Wieschaus EF, McGregor AP, Bialek W, Tank DW (2007) Stability and nuclear dynamics of the bicoid morphogen gradient. Cell 130: 141–152.

6. Abu-Arish A, Porcher A, Czerwonka A, Dostatni N, Fradin C (2010) High mobility of bicoid captured by fluorescence correlation spectroscopy: Implication for the rapid establishment of its gradient. Biophys J 99: L33–L35.

Table S2. The standard set of the most important parameters in our model.

Parameter / Quantity	Symbol	Value	Unit	Remarks
**Geometry**				
Number of nuclei in axial direction x	N_{x}	64		
Number of nuclei in circumferential direction ϕ	N_{ϕ}	64		
Internuclear distance (lattice spacing)	l	8.5	μm	supported by [2]
- resulting embryo length	L	544	μm	
Nuclear radius	R	6.5	μm	supported by [5]
- resulting nuclear volume	V	143.8	μm^3	assuming spherical shape
**Morphogen / activator gradients**				
Standard copy number at the poles	A_0	6720		varied in some simulations
- resulting copy number at half activation	$A_{\frac{1}{2}}$	690		supported by [2]
Morphogen diffusion length	λ	119.5	μm	supported by [2]
**Activation dynamics**				
Intranuclear activator diffusion constant	D_{A}	3.2	$\mu m^2/s$	supported by [6]
Activator binding site target size	α	10	nm	
- resulting activator binding rate	$k_{\mathrm{on}}^{\mathrm{A}}$	0.40	$\mu m^3/s$	diffusion limited, $k_{\mathrm{on}}^{\mathrm{A}} = 4\pi\alpha D_{\mathrm{A}}$
Activator unbinding rate	$k_{\mathrm{off,n}}^{\mathrm{A}}$	$410/6^n$	1/s	n = no. of bound activator molecules
Hill coefficient of activation	$\mathrm{n}_{\mathrm{max}}$	5		supported by [2]
**Gap gene dynamics**				
Gap protein monomer production rate	β_0	3.37	1/s	
Gap protein monomer degradation rate	μ_{M}	$3.37{\cdot}10^{-2}$	1/s	
Gap protein dimer degradation rate	μ_{D}	$3.37{\cdot}10^{-3}$	1/s	
Dimerization forward rate	$k_{\mathrm{on}}^{\mathrm{D}}$	0.80	$\mu m^3/s$	same as $2k_{\mathrm{on}}^{\mathrm{A}}$
Dimerization backward rate	$k_{\mathrm{off}}^{\mathrm{D}}$	$5.59{\cdot}10^{-3}$	1/s	
- predicted total Hb (Kni) copy number at full activation and $D = 0$	H_{β_0} (K_{β_0})	775		
Gap protein diffusion constant	D	varied		
**Repression dynamics**				
Repressing dimer binding rate	$k_{\mathrm{on}}^{\mathrm{R}}$	0.40	$\mu m^3/s$	same as $k_{\mathrm{on}}^{\mathrm{A}}$
Repressing dimer unbinding rate	$k_{\mathrm{off}}^{\mathrm{R}}$	$5.27{\cdot}10^{-3}$	1/s	equal to $0.1 \cdot k_{\mathrm{A},5}^{-}$; varied in some simulations

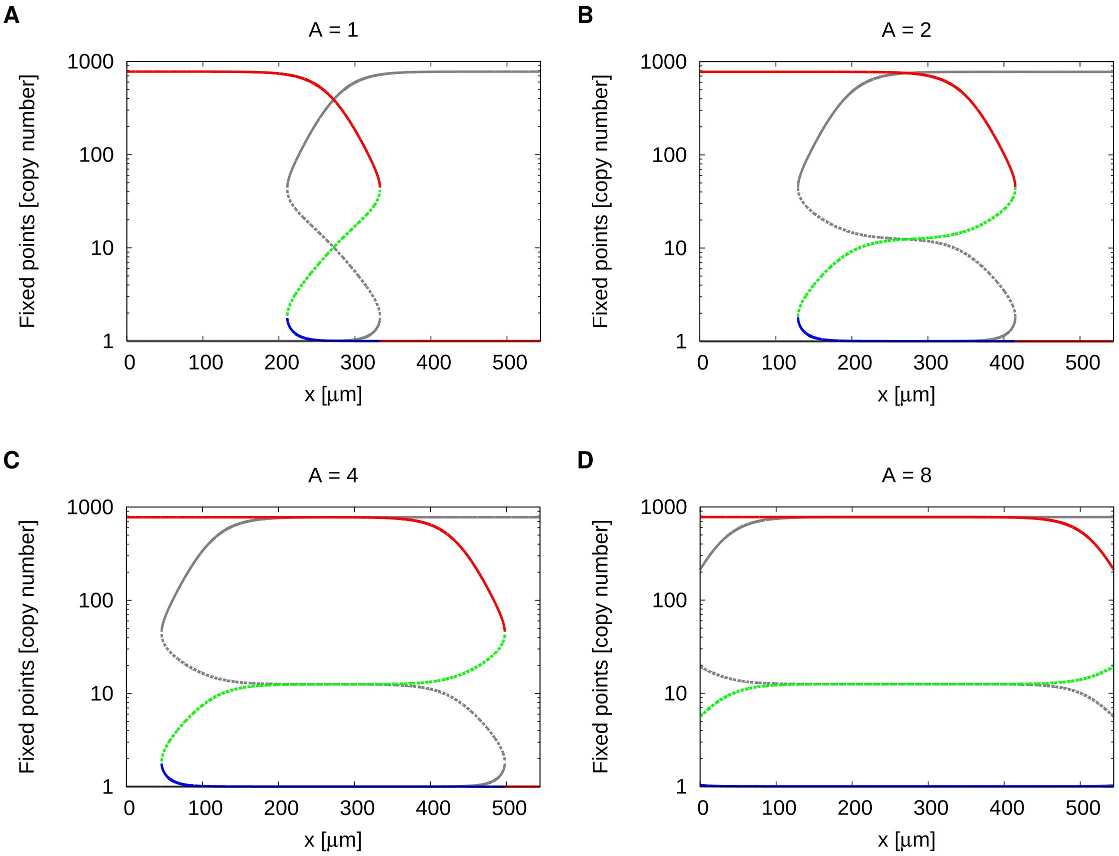

Figure S1. Bifurcation analysis of a one dimensional mean-field model of mutually repressing gap genes activated by morphogen gradients. Plotted are the stable (solid lines) and unstable (dashed lines) fixed points of the copy number of Hb (colored lines) and Kni (grey lines) as a function of the AP position x as predicted by a bifurcation analysis performed on a 1D mean-field model in which *hb* and *kni* are activated cooperatively by their respective morphogens and mutually repressing each other. Different colors correspond to different fixed points. The different panels show the solutions for activator amplitudes **(A)** A=1, **(B)** A=2, **(C)** A=4 and **(D)** A=8. All other parameters values are the standard values from Table S2. Activator concentrations at position x used in the mean-field analysis correspond to the ones in the 2D stochastic simulations. Away from midembryo each gap protein level has only one stable fixed point and one of the two levels is always zero. For all A there is a region around midembryo in which the protein levels have two stable and one unstable fixed point, implying bistability. In this region the analysis predicts bistable switching between the high-Hb–low-Kni and the low-Hb–high-Kni state. For clarity we color-code the Hb fixed points only. The Kni solutions are identical to the Hb solutions mirrored with respect to midembryo.

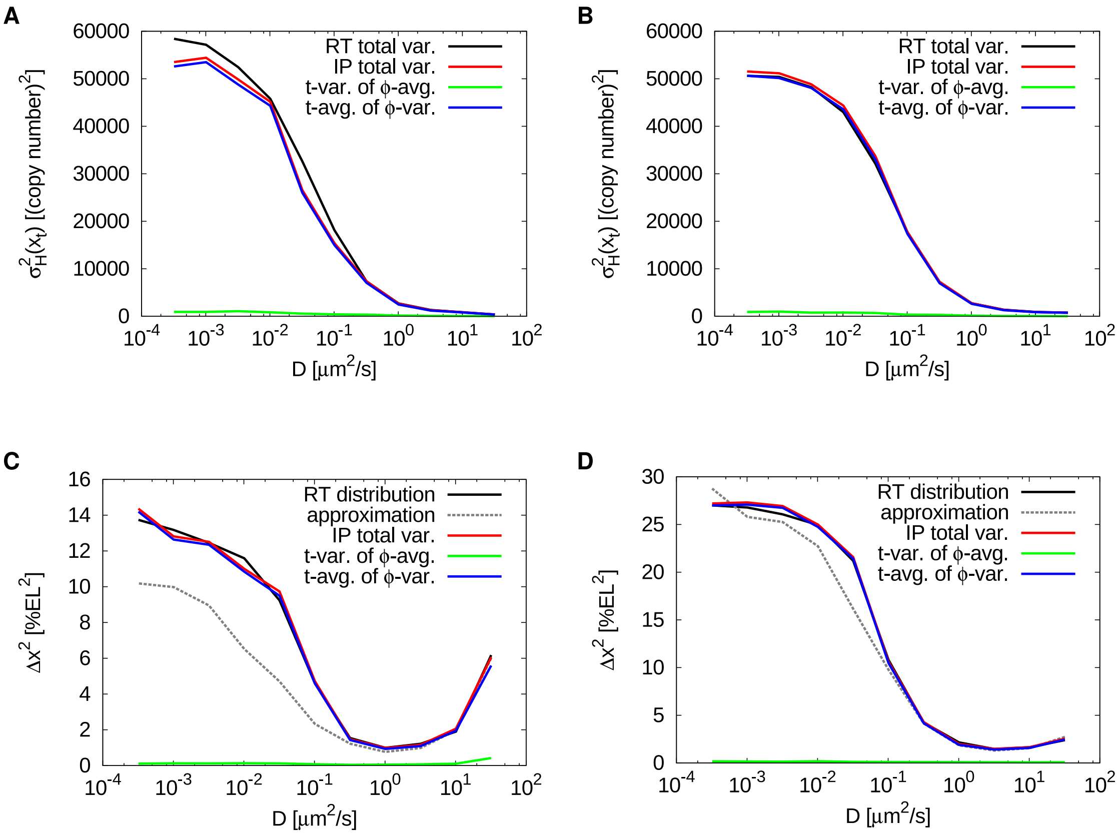

Figure S2. Decomposition of variances at the boundary. (A) Decomposition of the total Hb copy number variance at the average boundary position for the system with mutual repression as a function of the gap protein diffusion constant D. Plotted are: $\sigma^2_{\mathrm{H}}(x_{\mathrm{t}})$ the total (time- and circumference-) variance measured during runtime (RT, black), the same quantitity determined from a set of 6400 instantaneous profiles (IP, red, 64 AP rows at 100 different time points), $\sigma^2_{\langle \mathrm{H} \rangle_\phi}$ the variance in time of the circumference average of $H(x_{\mathrm{t}}, \phi)$ (green) and $\overline{\sigma^2_{\mathrm{H}}}$ the time average of the variance of $H(x_{\mathrm{t}}, \phi)$ over the circumference (blue). **(B)** The same as (A) for the system without mutual repression. **(C)** The same variance decomposition as in (A) for the Hb boundary position x_{t} instead of the copy number. The black line shows the Δx values measured as the standard deviation of the boundary position histogram accumulated during runtime (RT), the grey dashed line the corresponding values determined from the approximation $\sigma_{\mathrm{H}}(x_{\mathrm{t}})/|\langle H(x_{\mathrm{t}})\rangle'|$. **(D)** The same as in (C) for the system without mutual repression. In both cases, the main contribution to the total boundary variance $\sigma^2_{\mathrm{x_t}}$ comes from $\overline{\sigma^2_{\mathrm{x_t}}}$, implying that the blurring of the boundary is rather due to roughness than due to concerted boundary movements.

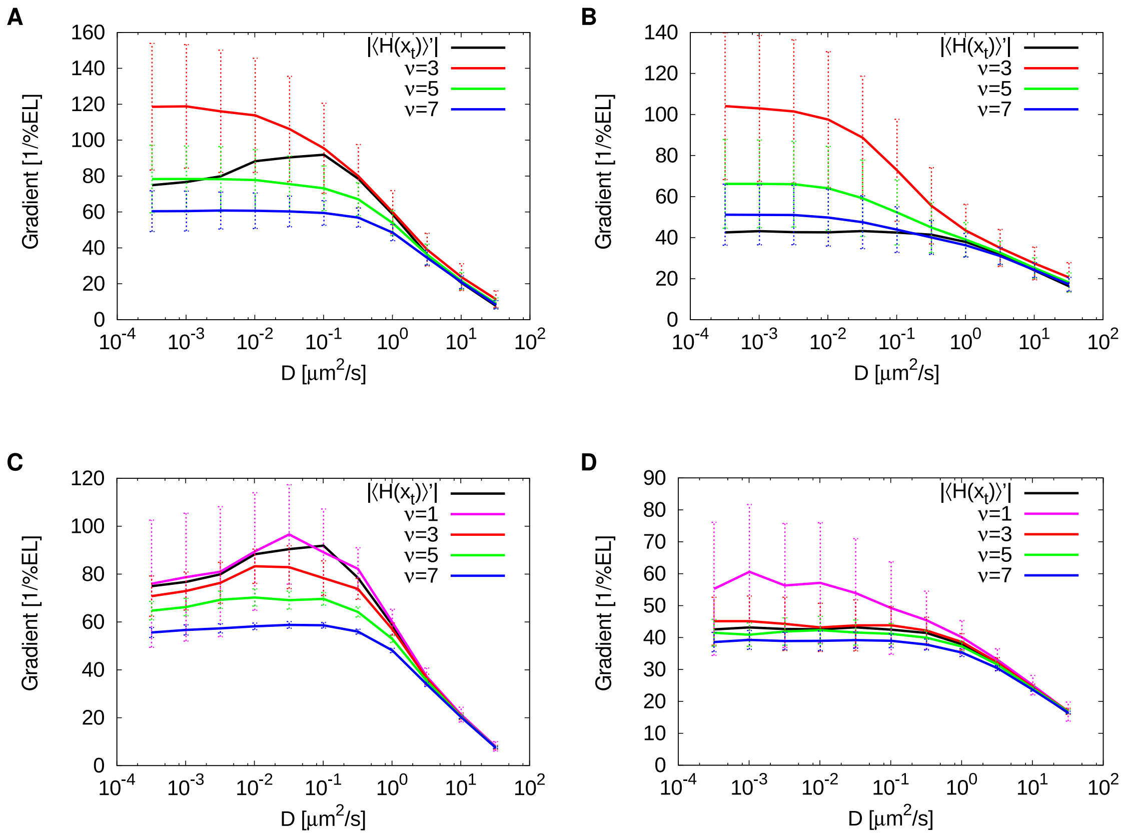

Figure S3. Microscopic properties of the boundary steepness. Panels **(A)** and **(B)** compare for different gap protein diffusion constants D the average Hb profile steepness at the boundary measured in a set of 6400 instantaneous profiles (64 AP rows at 100 different time points) to the steepness of the (time- and circumference-) average of the Hb profile ($|\langle H(x_{\rm t})\rangle'|$, black) for different numbers ν of neighboring data points used in calculating running averages over the instantaneous profiles for the system with (A) and without (B) mutual repression. Although for increasing ν the instantaneous profiles become less steep as a consequence of the smoothening, the values for $\nu = 3$ indicate that the profiles at a given row and time instance are slightly steeper than the average profile. In panels **(C)** and **(D)** we show results of the same analysis performed on the 100 circumference-averages of the instantaneous profiles, again for the system with (C) and without (D) mutual repression. Here $\nu = 1$ is the data obtained without calculating running averages (magenta). In both systems the steepness of the ϕ-averaged profiles agrees reasonably well with the steepness of the average profile $|\langle H(x_{\rm t})\rangle'|$.

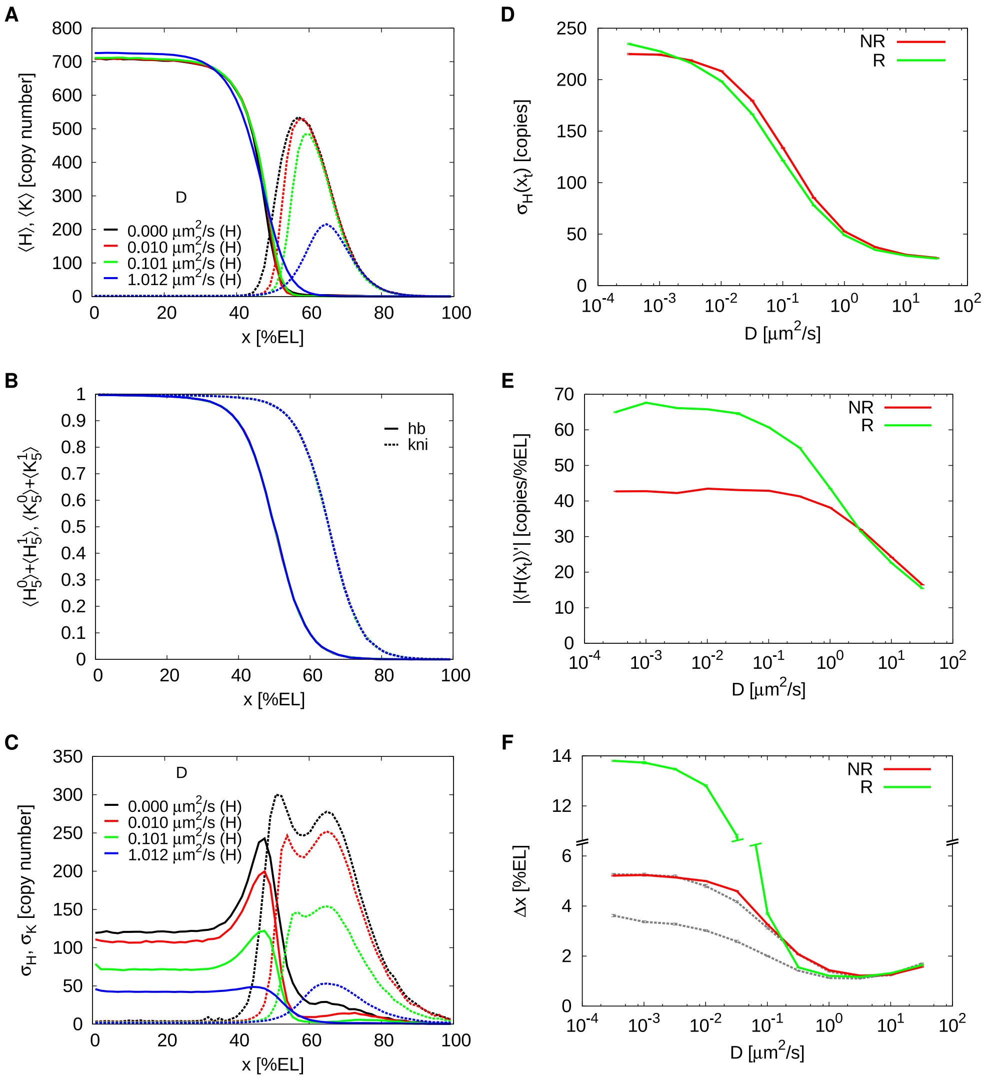

Figure S4. The effect of mutual repression in a system where both *hb* and *kni* are activated by the Bcd gradient. See following page for description.

Figure S4. The effect of mutual repression in a system where both *hb* and *kni* are activated by the Bcd gradient. (A) Time- and circumference-averaged Hb ($\langle H \rangle$, solid lines) and Kni ($\langle K \rangle$, dashed lines) total copy-number profiles along the AP-axis for various Hb and Kni diffusion constants D. **(B)** AP profiles of the average standard deviation of the total gap gene copy number for Hb (σ_{H}, solid lines) and Kni (σ_{K}, dashed lines). Note that the noise in K in the Kni domain is larger than that in H in the Hb domain. **(C)** AP profiles of the probabilities $\langle H_5^0 \rangle + \langle H_5^1 \rangle$ and $\langle K_5^0 \rangle + \langle K_5^1 \rangle$ that the *hb* (solid lines) and *kni* (dashed lines) promoters have 5 Bcd molecules bound to them, respectively; in the absence of mutual repression between *hb* and *kni*, these profiles would directly determine the expression of *hb* and *kni*. **(D)** The noise in the Hb copy number at the *hb* expression boundary as a function of the Hb and Kni diffusion constant D. **(E)** The steepness of the *hb* expression boundary as a function of the diffusion constant of the gap proteins. **(F)** The width Δx of the *hb* expression boundary as a function of the Hb and Kni diffusion constant. The grey line corresponds to the approximation $\Delta x \approx \sigma_{\mathrm{H}}(x_{\mathrm{t}})/|\langle H(x_{\mathrm{t}}) \rangle'|$, which we consider to be more reliable than Δx as measured from the distribution of threshold crossings, $p(x)$; the latter suffers from sporadic but strong suppression events of *hb* by *kni* in the anterior, which leads to a long tail of $p(x)$, increasing Δx. It is seen that while mutual repression has hardly any effect on the noise in the copy number at the boundary, it does markedly enhance the steepness of the boundary, and thereby its precision. The ratio of the Hb–*kni*-promoter dissociation rate over the Kni–*hb*-promoter dissociation rate is $k_{\mathrm{off}}^{\mathrm{R,H}}/k_{\mathrm{off}}^{\mathrm{R,K}} = 1/3$.

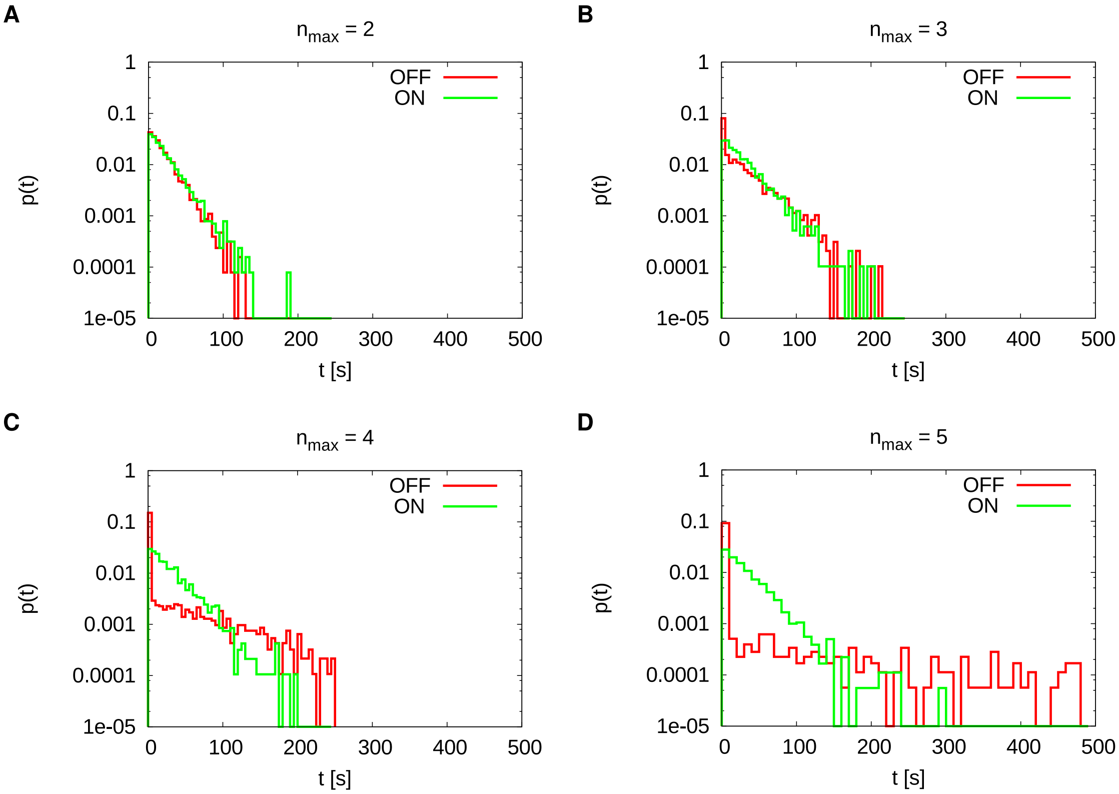

Figure S5. On- and off-times distributions of the *hb* promoter for different Hill coefficients n_{max} in a nucleus at midembryo. The panels show normalized histograms of the times spent in the producing ($n = n_{max}$) promoter state ("ON", green) and of the times spent in the non-producing ($n < n_{max}$) states ("OFF", red) for **(A)** $n_{max} = 2$, **(B)** $n_{max} = 3$, **(C)** $n_{max} = 4$ and **(D)** $n_{max} = 5$ (standard case). It can be seen that with increasing Hill coefficient n_{max} the off-times distribution changes from an exponential to a non-exponential distribution with high weight on very short off-times (implying fast returns to the producing state) and a with a long tail of long off-times. Since the off-rate from the producing state is kept the same for all n_{max} the on-times distributions remain unaltered. The on- and off-times have been determined from long time trajectories ($t_{\text{total}} = 10^5\ s$) of the occupancy of the producing state with a sampling resolution of $0.5\ s$.

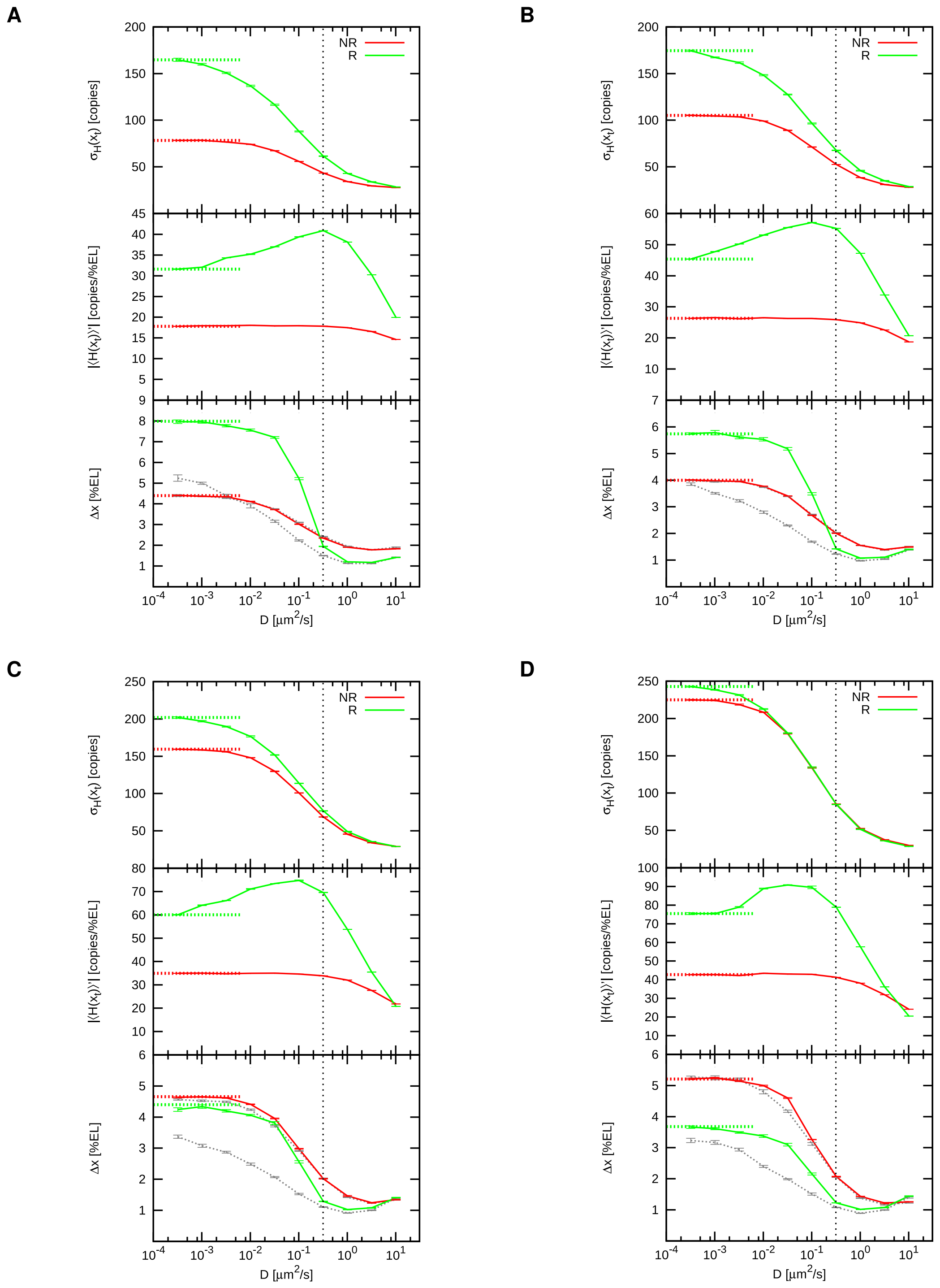

Figure S6. Boundary characteristics for reduced Hill coefficients n_{max}. See following page for description.

Figure S6. Boundary characteristics for reduced Hill coefficients n_{max}. The standard deviation of the total Hb copy number at the boundary ($\sigma_{\mathrm{H}}(x_{\mathrm{t}})$, upper panels), the gradient of the average Hb total copy number gradient at the boundary ($|\langle H(x_{\mathrm{t}})\rangle'|$, middle panels) and the boundary width (Δx, lower panels) as a function of the gap protein diffusion constant D for the systems with (green) and without (red) mutual repression and Hill coefficients **(A)** $n_{max} = 2$, **(B)** $n_{max} = 3$, **(C)** $n_{max} = 4$ and **(D)** $n_{max} = 5$ (standard case). Grey dashed lines are values determined from the approximation $\Delta x = \sigma_{\mathrm{H}}(x_{\mathrm{t}})/|\langle H(x_{\mathrm{t}})\rangle'|$, solid lines are values calculated from the distributions of x_{t}. Broad dashed lines are the values for $D = 0$. Black dotted lines mark the D-value where the boundaries are both steep and precise due to mutual repression.

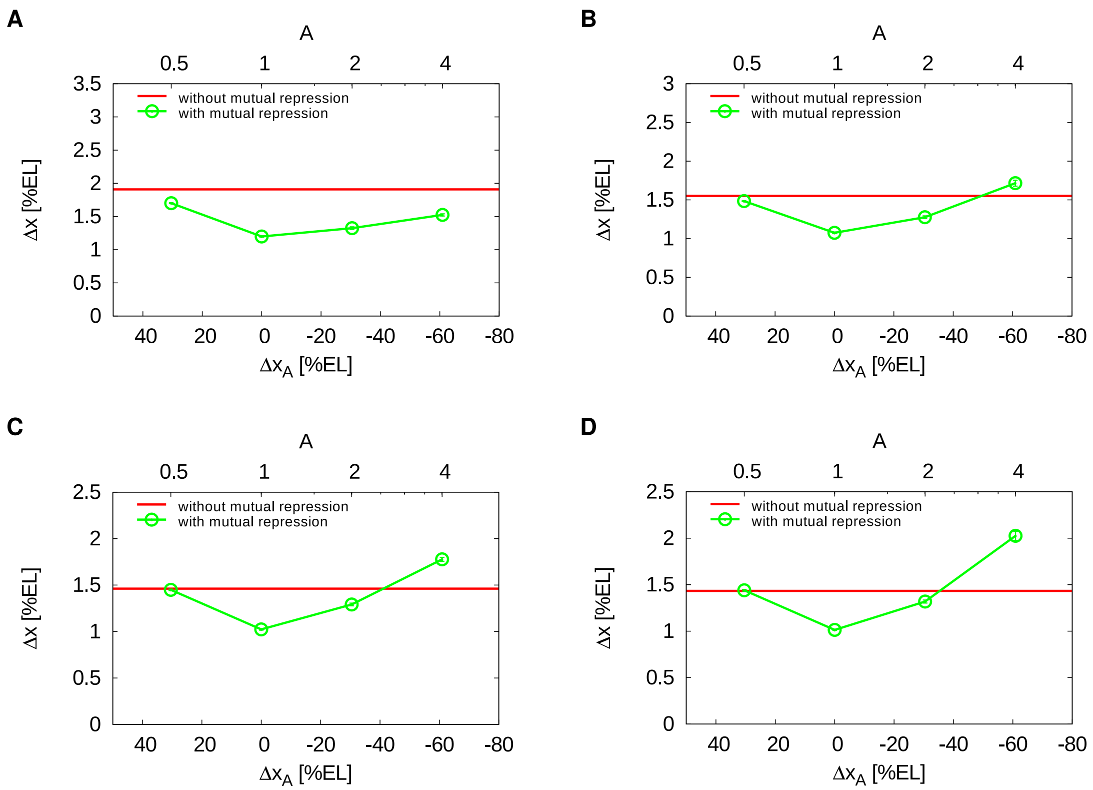

Figure S7. The effect of changing the activator amplitude A on the boundary precision for reduced Hill coefficients n_{max}. Shown are the the boundary width Δx with (green) and without (red) mutual repression as a function of Δx_{A}, the separation between the Hb and Kni boundaries expected in the system without mutual repression, and the corresponding activator amplitude A for Hill coefficients **(A)** $n_{max} = 2$, **(B)** $n_{max} = 3$, **(C)** $n_{max} = 4$ and **(D)** $n_{max} = 5$ (standard case). In all cases $D = 1.0\ \ \mu\mathrm{m}^2/\mathrm{s}$.

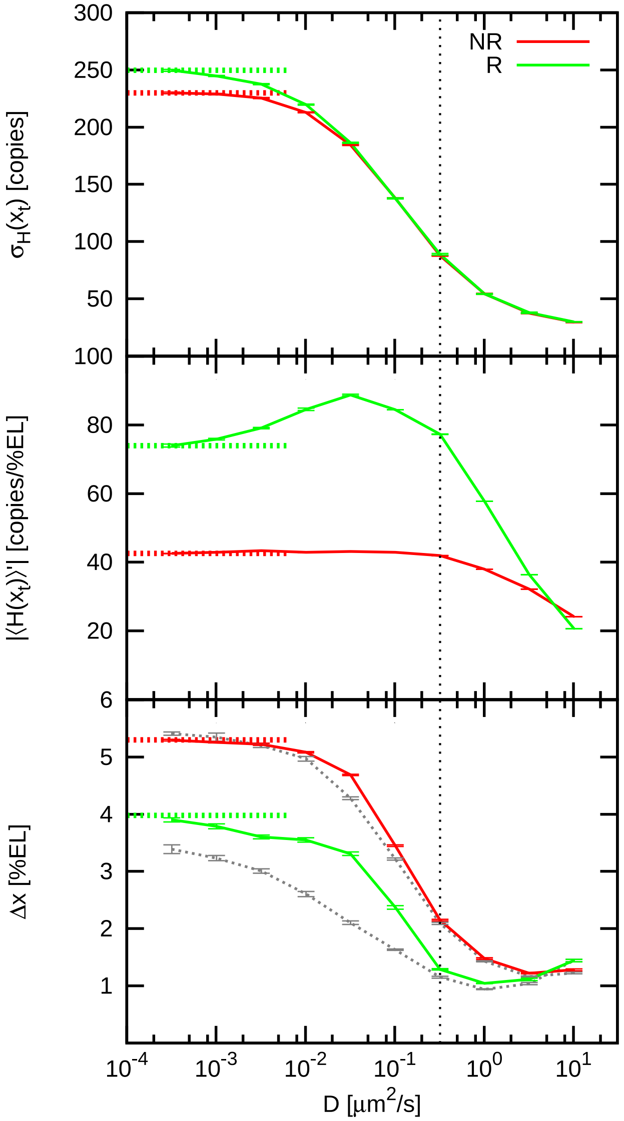

Figure S8. The effect of bursty gap protein production on the Hb boundary precision. The plot shows $\sigma_{\mathrm{H}}(x_{\mathrm{t}})$ the standard deviation of the total Hb copy number at the boundary, the steepness $|\langle H(x_{\mathrm{t}})\rangle'|$ of the average total Hb copy number profile at the boundary and the boundary width Δx with (green) and without (red) mutual repression as a function of the gap protein diffusion constant D for a system in which the gap proteins are produced in bursts of 10 at a time with decreased production rate $\beta = \beta_0/10$. The grey dashed lines are the values obtained from the approximation $\Delta x = \sigma_{\mathrm{H}}(x_{\mathrm{t}})/|\langle H(x_{\mathrm{t}})\rangle'|$. Thick dashed lines are values for $D = 0$. Error bars were obtained from block averages over 10 independent samples. The black dotted line marks the D-value where the boundary is both steep and precise due to mutual repression.

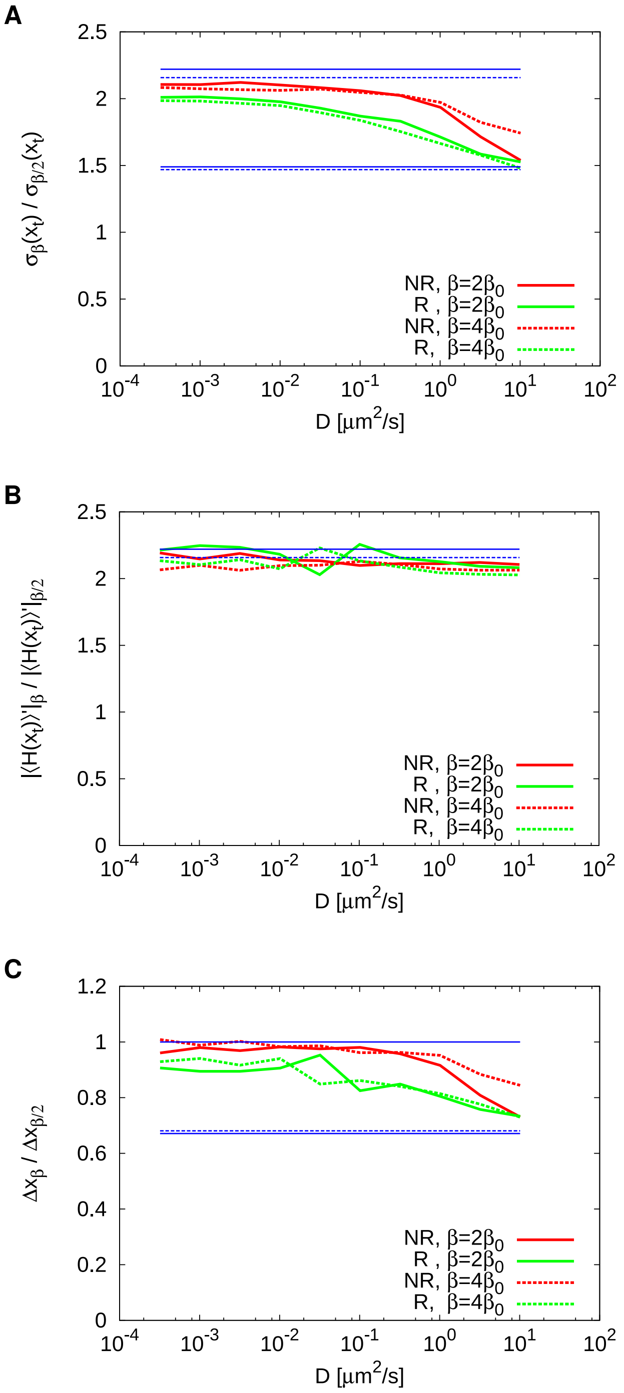

Figure S9. The effect of increased copy number on the Hb boundary precision. See following page for description.

Figure S9. The effect of increased copy number on the Hb boundary precision. Shown are the value ratios of important boundary properties for production rates $\beta > \beta_0$ as compared to $\beta/2$ for **(A)** the total Hb copy number noise $\sigma_{\mathrm{H}}(x_{\mathrm{t}})$ at the boundary, **(B)** the steepness of the average Hb profile at x_{t}, and **(C)** the resulting width Δx with (green) and without (red) mutual repression. Solid lines are for $\beta = 2\beta_0$, dashed lines for $\beta = 4\beta_0$. Blue lines depict the ratios as predicted from the expected scaling behavior for the limits of $D \to 0$ (upper line pairs) and $D \longrightarrow \infty$ (lower line pairs). The steepness is expected to scale precisely with the increased copy number in both limits. Note that the expected factor of copy number increase upon doubling β is not precisely two because of the nontrivial dependence of the monomer-dimer equilibrium on the production rate.
